# Protective effects of chlorogenic acid against LPS-induced intestinal oxidative injury in mice via activation of the PI3K/Akt-Nrf2/HO-1 signaling axis

**DOI:** 10.3389/fvets.2026.1870702

**Published:** 2026-07-10

**Authors:** Ying He, Yuhan Wu, Yue Wei, Yuan Wang, Caiping Feng

**Affiliations:** Department of Biological and Food Engineering, Lyuliang University, Lvliang, Shanxi, China

**Keywords:** animal nutrition, chlorogenic acid, feed additive, intestinal oxidative injury, molecular docking, network pharmacology, PI3K/Akt-Nrf2/HO-1 signaling axis

## Abstract

**Introduction:**

Oxidative stress is a key pathological cause of intestinal epithelial injury and related intestinal diseases. Chlorogenic acid (CGA), a natural polyphenol, has notable antioxidant activity. This study explored the multi-target protective effects of CGA on intestinal epithelial cells and mouse intestinal tissue against oxidative injury, as well as its underlying molecular mechanisms.

**Methods:**

Network pharmacology was applied to screen core targets and enriched pathways of CGA against oxidative stress. H₂O₂-induced MODE-K cell oxidative damage model and LPS-induced mouse intestinal injury model were constructed. CCK-8, biochemical detection, qPCR, molecular docking, Western blotting and histopathological assay were performed to verify the protective effects and related signaling pathways.

**Results:**

Network pharmacology predicted PI3K/Akt and Nrf2/HO-1 pathways as crucial targets. *In vitro*, CGA elevated cell viability, reduced oxidative injury markers, upregulated antioxidant factors, and regulated apoptosis-related proteins. CGA blocked Keap1-Nrf2 binding, activated Nrf2/HO-1 and promoted Akt phosphorylation. *In vivo*, CGA alleviated LPS-induced intestinal pathological injury, while PI3K inhibitor LY294002 reversed its protective effects, confirming the critical role of PI3K/Akt-Nrf2/HO-1 axis.

**Conclusion:**

CGA exerts multi-target antioxidant and intestinal protective effects by inhibiting Keap1-Nrf2 interaction and activating PI3K/Akt-Nrf2/HO-1 signaling axis, mitigating intestinal oxidative injury. Our findings provide a mechanistic basis for the application of CGA as a functional food ingredient or dietary intervention to alleviate intestinal oxidative stress in livestock.

## 1 Introduction

Oxidative stress is a common pathological condition afflicting livestock and poultry that are intensively farmed. Its causes may include environmental toxins, nutritional imbalances, or pathogenic infections, which can disrupt the balance between free radicals and antioxidants (1). Oxidative stress can damage the intestinal barrier and lead to inhibited growth and immunosuppression (2). Certain production diseases, such as ruminal acidosis in ruminants, weaning stress in piglets, and heat stress in poultry, are closely linked to oxidative damage to intestinal epithelial cells (3). As the primary barrier for nutrient absorption and microbial defense, the integrity of small intestinal epithelial cells directly influences an animal’s health. An excessive buildup of reactive oxygen species (ROS) can instigate intestinal cell apoptosis, the breakdown of tight junction proteins, and inflammatory factor storms, which can worsen production problems such as diarrhea and low feed conversion rates (4). The pursuit of safe and effective natural antioxidants to mitigate oxidative stress damage in livestock and poultry has become a crucial focus in the realm of animal nutrition.

Chlorogenic acid (CGA) is a potent polyphenolic compound abundant in plants used in animal feeds and traditional Chinese veterinary medicine. We selected CGA for this study based on three key points: its established safety as a natural feed additive, well-documented broad-spectrum biological activity, and the critical gap in understanding its precise mechanism of action against intestinal oxidative stress. Unlike synthetic antioxidants, CGA is natural, aligning with the growing consumer demand for antibiotic-free, natural animal production systems (5). Recent research shows CGA exerts significant intestinal protective effects in animal models by neutralizing free radicals, activating the Nrf2/ARE antioxidant pathway, and inhibiting the TLR4/NF-κB inflammatory cascade. Liu et al. demonstrated CGA reduces oxidative stress-induced injury and apoptosis; Hoseinynejad et al. found it mitigates inflammation and oxidative stress damage via free radical scavenging (6). Liu et al. further reported that CGA supplementation improves intestinal integrity, reduces oxidative stress and inflammation, and enhances growth performance in lipopolysaccharide-challenged broilers (7). However, most existing studies focus on single pathways or limited targets, primarily in inflammation models. A systematic, multi-target investigation of CGA’s protective mechanism against oxidative stress in small intestinal epithelial cells remains lacking. The uniqueness of our study lies in its comprehensive approach to deciphering this multi-target mechanism.

In recent years, network pharmacology has offered a new approach to investigating the multi-target mechanisms of natural products. By creating an “ingredient-target-pathway” interactive network, it is feasible to predict the key targets and pathways that CGA regulates in oxidative stress; such work can guide experimental verification. Huang et al. used network pharmacology to identify the core targets of non-alcoholic fatty liver disease and assessed the potential gene of cassia seed and the primary target in terms of binding affinity using molecular docking models. The team’s findings revealed that modulating six key genes and regulating the MAPK signaling pathway reduced the expression of CASP3 and EGFR. Moreover, low concentrations of cassia seed ethanolic extract significantly decreased lipid accumulation when treating lipid metabolism. This discovery provides theoretical and empirical foundations for further exploring the efficacy and mechanisms of traditional Chinese medicine in treating non-alcoholic fatty liver disease (8). Dai et al. used network pharmacology to examine the active ingredients and potential targets of Antidesma macrocarpon against colorectal cancer. By constructing PPI network maps, the team found that SRC (proto-oncogene tyrosine-protein kinase Src), MAPK1 (mitogen-activated protein kinase 1), ESR1 (estrogen receptor 1), HSP90AA1 (heat shock protein 90 alpha family class A member 1), and MAPK8 (mitogen-activated protein kinase 8) were the most strongly correlated with oxidative stress. Enrichment analysis further identified MAPK1 and MAPK8 as the key active constituents in Antidesma macrocarpon that mediate its anti-colorectal cancer activity. Additionally, the MTT method validated that an extract of Antidesma macrocarpon effectively inhibited the proliferation of HCT-116 and SW620 cells; the extract exhibited remarkable anti-colorectal cancer activity (9). Zhou et al. applied network pharmacology to explore the molecular mechanism of Shao Yao Gan Cao decoction in treating gastric cancer. Their findings suggested that this decoction inhibited the proliferation of gastric cancer cells, induced apoptosis, and promoted cell cycle arrest by regulating the PI3K/Akt and MAPK signaling pathways, while also reducing cloning capacity (10). This methodology is particularly suited for CGA, as its efficacy is likely mediated via interactions with multiple targets rather than a single one. By employing this approach, we move beyond the conventional single-target perspective to build a holistic understanding of the mechanisms of action of CGA. Such work represents a significant advancement over existing studies.

Although prior research has established the antioxidant capacity of CGA, this work provides the first comprehensive analysis, integrating network pharmacology with systematic experimental assays, to elucidate its multi-target actions in intestinal epithelial cells from computational prediction to functional confirmation. Here, we combine network pharmacological predictions with rigorous *in vitro* validation to systematically elucidate the multi-target protective mechanisms of CGA against oxidative stress in mouse small intestinal epithelial (MODE-K) cells. This integrated strategy allows us to not only confirm the efficacy of CGA but also to map the entire network of interactions—from key targets to signaling pathways—that mediates its antioxidant effects. We hypothesize that CGA activates a coordinated cellular defense program against oxidative stress by modulating core targets within the PI3K/Akt and Nrf2 signaling pathways. This research provides a comprehensive theoretical foundation for the application of CGA as a targeted nutritional intervention to improve intestinal health in livestock production.

Further *in vivo* animal experiments revealed that LPS-induced small intestinal injury was accompanied by significant intestinal barrier disruption, aggravated inflammatory infiltration, and increased apoptosis of intestinal epithelial cells. Meanwhile, chlorogenic acid (CGA) could significantly alleviate these pathological damages. To systematically clarify its regulatory mechanism, this study combined network pharmacology prediction with experimental verification to deeply explore the mechanism by which CGA protects intestinal epithelial cells from oxidative stress damage through a multi-target molecular mechanism. This study can provide a theoretical basis for the application of chlorogenic acid as a functional nutritional additive in the regulation of intestinal health and antioxidant nutrition in livestock and poultry, and also provides a more comprehensive theoretical support for its application as a natural antioxidant in animal husbandry production.

## 2 Materials and methods

### 2.1 Reagents and drugs

CGA (purity ≥ 98%) was purchased from Shanghai Yuanye Biological Technology Co., Ltd. (Shanghai, China). The RPMI-1640 medium and PS were procured from Gibco (Grand Island, NY, USA). Fetal bovine serum (FBS) came from Zhejiang Shuanghua Biological Technology Co., Ltd. (Hangzhou, China). Trypsin and BCA protein quantification kits were sourced from Wuhan Boster Biological Technology Co., Ltd. (Wuhan, China). PBS was obtained from Beijing Solarbio Science & Technology Co., Ltd. (Beijing, China). CCK-8 assay kits, ROS detection kits, and Annexin V-FITC apoptosis detection kits were procured from Shanghai Biyuntian Biological Technology Co., Ltd. (Shanghai, China). Superoxide Dismutase (SOD), Catalase (CAT), glutathione (GSH), Lactate Dehydrogenase (LDH), Glutathione Peroxidase (GSH-Px) and Malondialdehyde (MDA) assay kits were acquired from Shanghai Enzyme-Linked Biotechnology Co., Ltd. (Shanghai, China). RNAiso Plus, PrimeScript™ RT reagent Kit with gDNA Eraser, and TB Green® Premix Ex Taq™ II (Tli RNaseH Plus) were purchased from Baoji Medical Biotechnology Co., Ltd. (Beijing, China). Chloroform, isopropanol, and absolute ethanol were obtained from Chengdu Kelong Chemical Co., Ltd. (Chengdu, China), Tianjin Kaitong Chemical Reagent Co., Ltd. (Tianjin, China), and Tianjin Fengchuan Chemical Technology Co., Ltd. (Tianjin, China) respectively. Agarose powder was purchased from Invitrogen (Carlsbad, CA, USA). Primary antibodies against Nrf2, Keap1, HO-1, *β*-actin, Bcl-2, Bax, cleaved-Caspsae-3, Akt, and phosphorylated Akt (p-Akt), as well as secondary antibodies, were acquired from Wuhan Sanying Biotechnology Co., Ltd. (Wuhan, China). LY294002 was purchased from Shanghai Biotechnology Engineering Co., Ltd. Acridine orange, ethidium bromide, and other analytical-grade chemical reagents were purchased from Sinopharm Chemical Reagent Co., Ltd. (Shanghai, China).

### 2.2 Network pharmacology analysis

#### 2.2.1 Screening of potential targets for CGA

The UniProt and ChemSpider databases are integral to research on chemical structures and literature. This study extracted the target structure and name of CGA from these two resources (11). Using these target names and structures, the corresponding SMILES encoding in the PubChem database was subsequently searched. The SMILES encoding analysis allowed for the prediction of targets matching the active ingredients. Following prediction, search, and deduplication processes, the target proteins associated with CGA were ultimately identified (12).

#### 2.2.2 Screening of oxidative stress-related targets

Using “oxidative stress” as a keyword, relevant targets were searched in CTD^1^, GeneCards^2^, and DisGeNET^3^ databases (13).

#### 2.2.3 Intersection target screening

Chlorogenic acid and its oxidative stress targets were input into the Venny 2.1.0 website to identify their overlapping targets related to oxidative stress, and corresponding Venny maps were drawn (14).

#### 2.2.4 Construction of PPI network

To determine the primary action target of CGA, predicted data related to proteins were initially imported into the SWISS Target Prediction database (15), with “*Mus musculus*” chosen as the screening species for constructing the PPI network. Subsequently, targets involved in oxidative stress-induced by CGA were incorporated into the STRING database (16). Only highly reliable targets with an interaction score of no less than 0.700 were selected. Unconnected nodes were hidden in the analysis results. The results were then visualized in the PPI network using Cytoscape 3.9.0 software (17). When selecting primary target points, topological characteristics of the protein–protein interaction network should be analyzed using the ‘Network Analyzer’ plugin in Cytoscape 3.9.0 software (18). This allows for the visualization of the protein network. Targets are only considered key when the Degree and Betweenness indicators exceed the average value (19).

#### 2.2.5 KEGG pathway enrichment analysis

The DAVID database is a potent tool predominantly utilized for gene function annotation and pathway enrichment analysis (20, 21). This Sankey diagram conducts KEGG annotation analysis using the selected core targets (Degree>5), and depicts the top 10 most significantly enriched KEGG pathways. After completing these analyses, the KEGG pathway map was drawn using the Wei sheng xin online platform^4^ (*p* < 0.05).

### 2.3 Molecular docking

Firstly, obtain the receptor file 2FLU, which corresponds to the Kelch region of the Keap1 protein, from the PDB protein database. Import this file into the Autodock software. Once inside the Autodock software environment, carry out rigid-body identification for the ligands. During this phase, set the rotatable bonds crucial for future calculations and analysis. After doing so, save the related files in pdbqt format.

Next, create the docking files for the ligand CGA, ensuring that the docking procedures are consistent between CGA and Keap1. Then, employ the grid program in Autodock software to calculate the grid points of the receptor and ligand. A grid box was set at the Kelch region of the key Keap1 protein, with the following coordinates: center_x = 5, center_y = 9, center_z = −1. The size of this grid box was set to 60 × 60 × 60 angstroms, and the grid spacing was 0.375 angstroms, to cover the entire binding pocket. The goal of these adjustments is to entirely encompass the ligand, and save the content with these adjustment details in a gpf format file.

Lastly, carry out the docking process using the genetic algorithm and the principle of minimizing docking energy. Once all docking operations are completed, analyze the three-dimensional and two-dimensional interactions between the receptor and ligand using the OpenBabel 2.3.1 software (22).

### 2.4 *In vitro* cell experiments

#### 2.4.1 Cell culture

The mouse small intestinal epithelial (MODE-K) cell line (CVCL_B4FG) was purchased from the Shanghai Guandao Biotechnology Engineering Co., Ltd. After removing the small intestinal epithelial cells from the CO₂ incubator, the culture medium is first aspirated. Next, an appropriate amount of digestion solution is added and left to stand for a while. Then, the digestion solution is removed, and a fresh culture medium is added. The cell suspension is prepared using the bubble method, centrifuged, the supernatant discarded, and the cells are resuspended in the culture medium. After resuspension, cells are incubated at 37 °C with 5% CO₂. When the cell coverage on the base of the culture dish reaches 80–90%, observations are made using an inverted microscope. Then, cells in the logarithmic growth phase are selected for subculture in subsequent experiments (23). All experiments were conducted with cells between passages 5 and 10 to ensure phenotypic stability and avoid senescence-related artifacts.

#### 2.4.2 CGA cytotoxicity assay (CCK-8 method)

In each well of a 96-well plate, 1 × 10^4^ cells were seeded. Once the cell density reached 80%, the old culture medium was first discarded, followed by the addition of a culture medium containing CGA, with final concentrations of 0 (control group), 12.5, 25, 50, 100, and 200 μmol/L. Each concentration group included three parallel samples. Subsequently, the samples were incubated in a culture incubator at 37 °C with a CO₂ concentration of 5% for 12 h. The cell viability was then measured (24).

#### 2.4.3 Cell model construction and drug administration

Cells in the logarithmic growth phase were used for the experiment. The control group, labeled “Control,” cultured cells in a normal culture medium without any additional treatment. The injury group, referred to as “Model,” added 150 μmol/L H₂O₂ to the cell culture after 4 h of treatment (25). The CGA (CGA + H₂O₂) treatment group cultured cells in normal culture medium for 24 h, and then introduced different concentrations of CGA (12.5, 25, 50, 100, and 200 μmol/L) for 12 h of treatment (26). Finally, cells from each group were collected for subsequent experimental analysis.

#### 2.4.4 Cell viability assay

In the experiment, mouse small intestinal epithelial cells were seeded into 96-well plates at a density of 1 × 10^4^ cells per well. After the grouping and drug treatment operations were completed according to section 2.4.3, 10 μL of the CCK-8 reagent was added to each well. This was followed by incubation at 37 °C for 2 h. Absorbance was measured after the incubation period (27). The cells were also counted by trypan blue staining.

#### 2.4.5 Detection of intracellular reactive oxygen species (ROS) levels

The mouse small intestinal epithelial cells were seeded into 96-well plates at a density of 1 × 104 cells per well and were grouped and treated with drugs as described in Section 2.3. Subsequently, following the instructions of the kit, 100 μL of working medium containing the DCFH-DA fluorescence probe was added to each well. After incubating at 37 °C for 20 min, the cells were rinsed twice with PBS, the intracellular ROS levels were measured using the BioTek Synergy H1 microplate reader (28).

#### 2.4.6 Detection of antioxidant/oxidative damage indicators

Cells were seeded into 6-well plates at a density of 2 × 105 cells per well. After inoculation, the cell culture medium was collected and centrifuged at 12,000 rpm for 10 min. The supernatant was then removed to determine the activity of LDH (29). Following this, the cells were digested with 0.05% EDTA trypsin and then centrifuged at 1000 rpm for 10 min to remove the supernatant and gather the cells. Subsequently, PBS was added to prepare the cell suspension. The cells were then disrupted using a cell disruptor to determine the content of MDA and the activities of SOD, CAT, GPx, and GSH (30). The determination of MDA, LDH, SOD, CAT, GPx, and GSH was conducted according to the kit instructions provided by the Shanghai Enzyme-linked Biotechnology Co., Ltd.

#### 2.4.7 Cell apoptosis detection

Mouse intestinal epithelial cells (2 × 105) were seeded per well in a 6-well plate, divided into groups, and treated with drugs as per section 2.4.3. Vc was used as a positive control. After this, apoptosis was evaluated according to instructions utilizing the FITC-Annexin-V and propidium iodide staining kit. Specifically, the MODE-K cells were treated with trypsin and washed twice with 1X PBS solution. Following centrifugation, the cells were gently resuspended in a tube containing 195 μL of the Annexin-V-FITC binding solution. Next, 5 μL of Annexin-V-FITC and 10 μL of PI were added to the tube. The cells were then allowed to incubate at room temperature in the dark for 15 min. Lastly, the cells underwent flow cytometry analysis using the Beckman Coulter CytoFLEX flow cytometer (31–33).

#### 2.4.8 Western blot test

After completing the grouping and drug treatment as per the provisions of Section 2.4.3, and adding a LY294002 group, CGA was first subjected to the same treatment. Then, using the pre-prepared LY294002 + H₂O₂ solution of the same volume, it was treated for 12 h. After the operation, the experiment employed a low-temperature cell lysis method, lysing cells in a pre-chilled lysis buffer infused with protease and phosphatase inhibitors. We collected the supernatant post-centrifugation. The protein concentration was determined using the BCA kit, with a BSA standard establishing a standard curve. We detected the absorbance at 562 nm by using an enzyme-linked instrument (34). After boiling protein samples for denaturation, we carried out an SDS-PAGE electrophoresis analysis with 12 and 10% separating gels. The initial electrophoresis ran for 30 min at 80 V, which we then adjusted to 120 V to prolong the electrophoresis for another 100 min. Subsequently, we transferred proteins to PVDF membranes using a wet transfer method, employing a consistent current of 200 mA for 1.5 h. Following the transfer, we blocked the membranes with 5% skim milk for 2 h and sequentially incubated them with primary and secondary antibodies. We incubated the primary antibody overnight at 4 °C, followed by the secondary antibody for 1 h at room temperature. After each antibody incubation, we thoroughly washed the membranes in TBST buffer. Finally, we stained the samples with a development solution, capturing images with a gel imaging system. We quantitatively analyzed protein bands using ImageJ software. Careful attention was required to maintain a low-temperature operational environment during the experiment to ensure the accuracy of protein quantification and prevent the formation of bubbles during the transfer process (35).

### 2.5 *In vivo* animal experiments

#### 2.5.1 Animals and experimental design

All animal procedures were approved by the Institutional Animal Care and Use Committee of Lyuliang University (Approval No.: LLXYLL20250401, 15 April 2026).

SPF-grade C57BL/6 male mice (6–8 weeks old, weighing 20 ± 2 g) were purchased from Beijing Vitogen Life Science Co., Ltd. They were housed in an SPF-grade animal room (12-h light–dark cycle, temperature 22 ± 2 °C, humidity 50 ± 5%) and were given an adaptive feeding for 7 days before being randomly divided into 4 groups, with 6 mice in each group. They were then fed under the same conditions:

The normal control group (CON group): 14 consecutive days, intragastric administration of equal volume of normal saline daily. On the 15th day, intraperitoneal injection of equal volume of normal saline was performed. The mice were sacrificed 24 h later.

The model control group (MOD group): 14 consecutive days, intragastric administration of equal volume of normal saline was performed daily. On the 15th day, 50 mg/kg LPS was injected intraperitoneally to establish a small intestine injury model. The mice were sacrificed 24 h after the model was established.

The CGA group (CGA group): 100 mg/kg CGA was administered intragastrically for 14 consecutive days (the optimal dose determined in the pre-experiment), and 50 mg/kg LPS was injected intraperitoneally on the 15th day. The mice were sacrificed 24 h after the injection.

The LY294002 group (LY294002 group): 100 mg/kg chlorogenic acid (CGA) was administered by gavage for 14 consecutive days, and then 10 mg/kg LY294002 was injected intraperitoneally on the 15th day (A single intraperitoneal injection was used, as previous studies have shown that LY294002 does not cause significant intestinal damage or oxidative stress in normal mice at this dose). One hour later, 50 mg/kg lipopolysaccharide (LPS) was injected intraperitoneally again, and the mice were sacrificed after the injection.

The weight of the mice is recorded every day. The Disease Activity Index (DAI) is evaluated based on the weight loss, fecal characteristics, and hematochezia. At the end of the experiment, all mice were euthanized by trained personnel using a rapid cervical dislocation (vertebral dislocation) method. No anesthetics were used in this study to avoid interference from drugs on intestinal oxidative stress and related molecular indicators. After euthanasia, the mice were immediately dissected. The small intestine tissues were quickly isolated; some of the tissues were fixed with 4% paraformaldehyde, while the rest were instantaneously frozen in liquid nitrogen and stored at −80 °C for subsequent analysis.

#### 2.5.2 Microscopic tissue section of the small intestine (H&E staining)

The small intestinal tissues were stained with HE to observe the pathological features, including mucosal morphology, villus structure and inflammatory infiltration. The removed mouse small intestinal tissues were placed in 4% paraformaldehyde fixative solution and fixed for 24–48 h. After fixation, the tissues were dehydrated successively with gradient ethanol, then stained with xylene, and fully immersed in paraffin to complete tissue embedding. Using a paraffin sectioning machine, the embedded tissue blocks were continuously cut into 4–5 μm thick sections, and the sections were spread, dried and stored for later use. The prepared paraffin sections were dehydrated, hydrated with gradient ethanol, and stained with hematoxylin–eosin (H&E) after staining. After staining, the sections were dehydrated, transparentized, and sealed with neutral gum to form permanent pathological sections. The mucosal morphology, villus structure, crypt morphology and epithelial cell integrity of the small intestinal tissues were observed under an optical microscope. The small intestinal tissue sections were quantitatively analyzed using Image-Pro Plus image analysis software. Randomly selected fields were measured the height of the villi (VH) and the depth of the crypts (CD). Multiple fields were selected for each sample to ensure data accuracy. Finally, the ratio of villus height to crypt depth (VH/CD) was calculated as an important indicator for evaluating the integrity of the small intestinal mucosa structure and the repair of damage.

#### 2.5.3 Immunohistochemical staining

The paraffin sections of small intestinal tissues were placed in a constant temperature box for baking, then dewaxed with xylene and hydrated through a gradient of ethanol to distilled water for rinsing, completing the pretreatment of the sections. Antigen retrieval was performed using citrate buffer under high temperature and high pressure, and the sections were naturally cooled to room temperature. They were then incubated in a 3% hydrogen peroxide solution in the dark to block endogenous peroxidase activity and eliminate non-specific background staining. Subsequently, normal goat serum blocking solution was added and incubated at room temperature to reduce non-specific binding. The blocking solution was discarded, and the sections were incubated with appropriately diluted primary antibody working solution in a wet box at 4 °C overnight. The next day, the sections were taken out and rewarmed, washed thoroughly with PBS buffer, and then incubated with secondary antibody working solution at room temperature. DAB chromogenic solution was used for chromogenic reaction in the dark, and the degree of chromogenic reaction was controlled under an optical microscope. When the chromogenic reaction was moderate, the reaction was terminated with distilled water. The sections were counterstained with hematoxylin for cell nuclei, dehydrated through a gradient of ethanol, cleared with xylene, and mounted with neutral resin. The positive expression of the target protein in the small intestinal tissues was observed under an optical microscope and images were collected. ImageJ image analysis software was used to quantitatively analyze the immunohistochemical images. Representative fields of view from each group of sections were randomly selected, and the integrated optical density (IOD) values of the positive staining areas were measured to objectively reflect the expression level of the target protein in the small intestinal tissues.

#### 2.5.4 Western blot detection of small intestinal tissue

Take an appropriate amount of small intestine tissue samples and place them in a pre-cooled mortar. Add RIPA lysis buffer containing phosphatase inhibitors and protease inhibitors, and grind thoroughly on ice until the tissue is uniformly disrupted. Transfer the lysate to a centrifuge tube and incubate on ice for 30 min, gently shaking it every 5 min. Centrifuge at 4 °C and 12,000 r/min for 15 min. Carefully aspirate the supernatant, which is the total protein sample. Use a BCA protein quantification kit to determine the protein concentration. Based on the quantification result, add an appropriate amount of 5 × SDS loading buffer, mix well, and boil in a water bath for 10 min to fully denature the protein. After a brief centrifugation, store at −20 °C for future use. Prepare the corresponding concentration of separation gel and stacking gel. Take an equal amount of protein samples for SDS-polyacrylamide gel electrophoresis (SDS-PAGE), and run the gel at a constant voltage until the bromophenol blue indicator reaches the bottom of the gel. Use the wet transfer method to transfer the protein from the gel to the PVDF membrane. After the transfer is complete, block the membrane with 5% skim milk at room temperature for 1.5 h. Cut the membrane strips according to the molecular weight of the target protein, add the primary antibody dilution solution, and incubate slowly on a 4 °C shaker overnight. The next day, wash the membrane three times with TBST washing solution for 10 min each. Then add the corresponding secondary antibody dilution solution and incubate at room temperature on a shaker for 1 h. Wash the membrane three times again with TBST for 10 min each. Use an ECL chemiluminescence kit for imaging, and collect the images using a gel imaging system and perform gray value analysis.

### 2.6 Statistical analysis

The data statistics and analysis were conducted using SPSS 23.0 software. One-way analysis of variance (ANOVA) was performed to compare differences among groups, followed by Tukey’s honest significant difference (HSD) *post hoc* test for multiple comparisons. GraphPad Prism 10.0 software was used for all graphs and plots and *p* < 0.05 was considered significant.

## 3 Results

### 3.1 The screening results (number) of potential targets and oxidative stress-related targets of CGA

**Figure 1:**
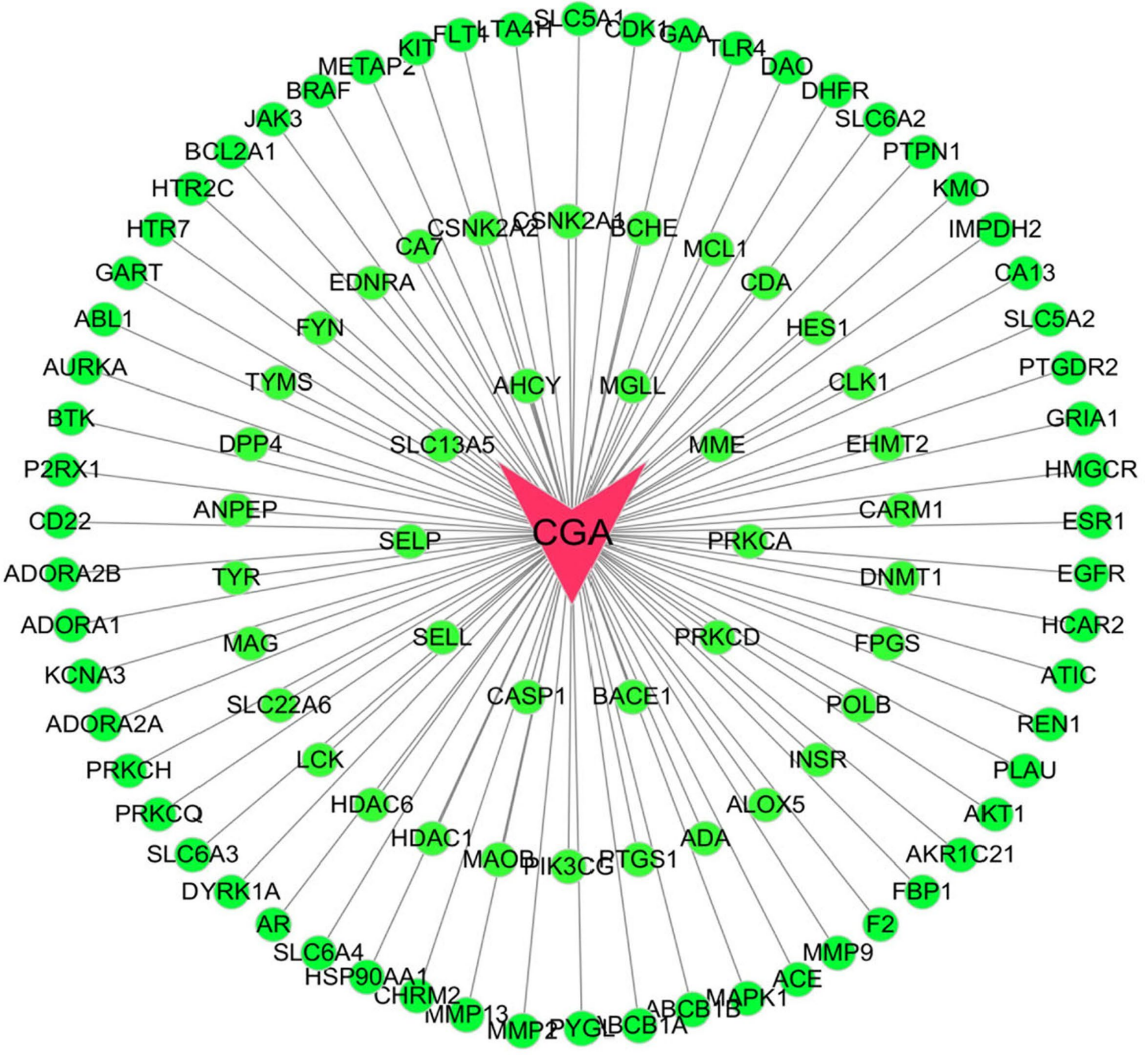
Potential target network of CGA.

We searched the SMILES code in the PubChem database for CGA and input the results into the SWISS Target Prediction database to identify related targets. We ultimately obtained 100 potential targets (Figure 1).

### 3.2 Intersection target screening results (Venn diagram, 81 intersection targets)

**Figure 2:**
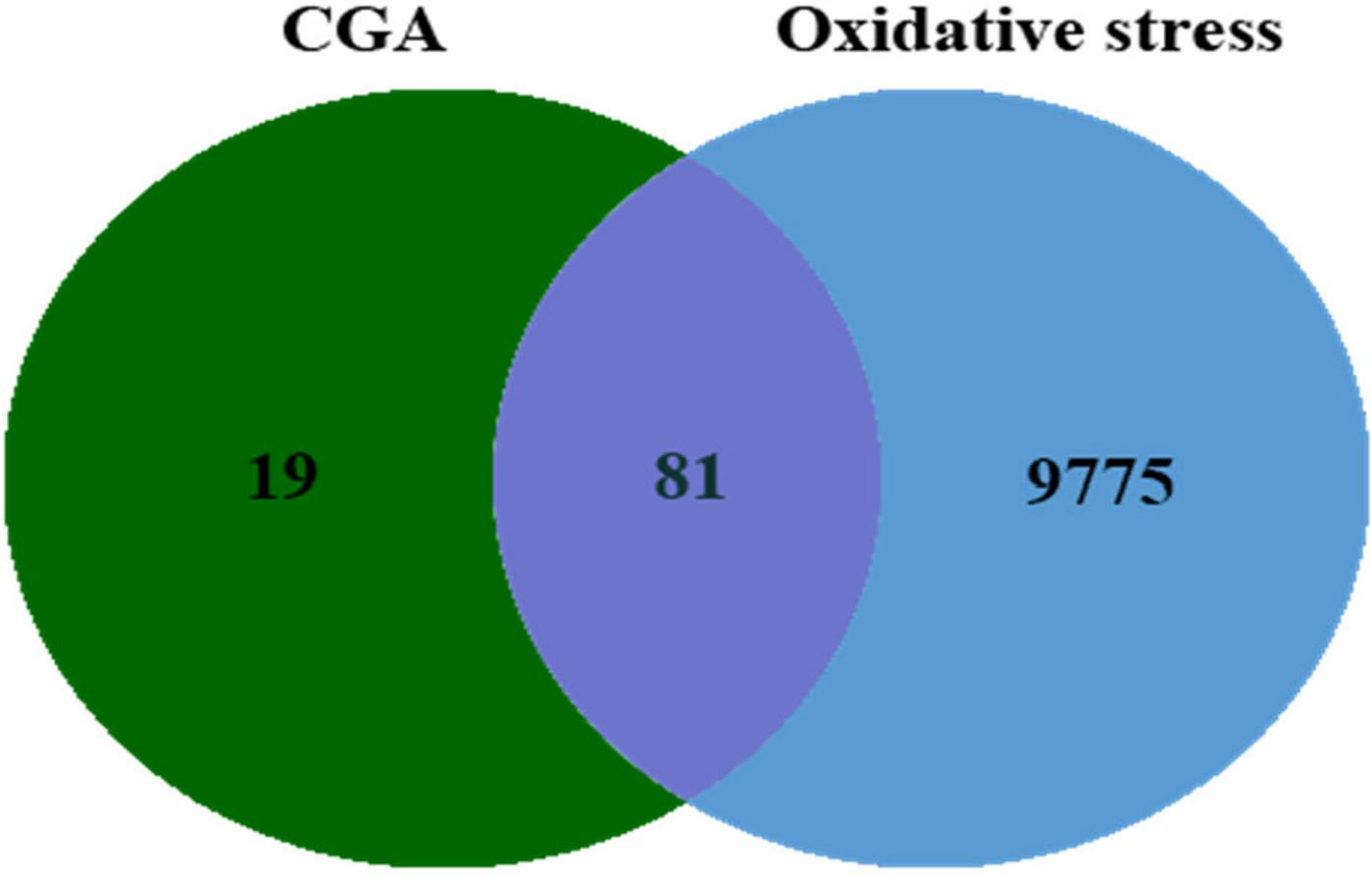
Venny diagram of the intersection targets between CGA and oxidative stress.

According to the GeneCards database, there are a total of 9,859 oxidative stress-related targets; 6,585 remain after median filtering. The OMIM database identifies 295 oxidative stress targets, resulting in 9,856 unique targets after de-duplication. When compared with the Venn diagram analysis of drug targets noted in Section 3.1, we find 81 intersecting target points (Figure 2).

### 3.3 Construction of PPI network and screening of core targets (network diagram, topological parameters, top 10 core targets: Hsp90aa1/kit/Fyn, etc.)

**Figure 3:**
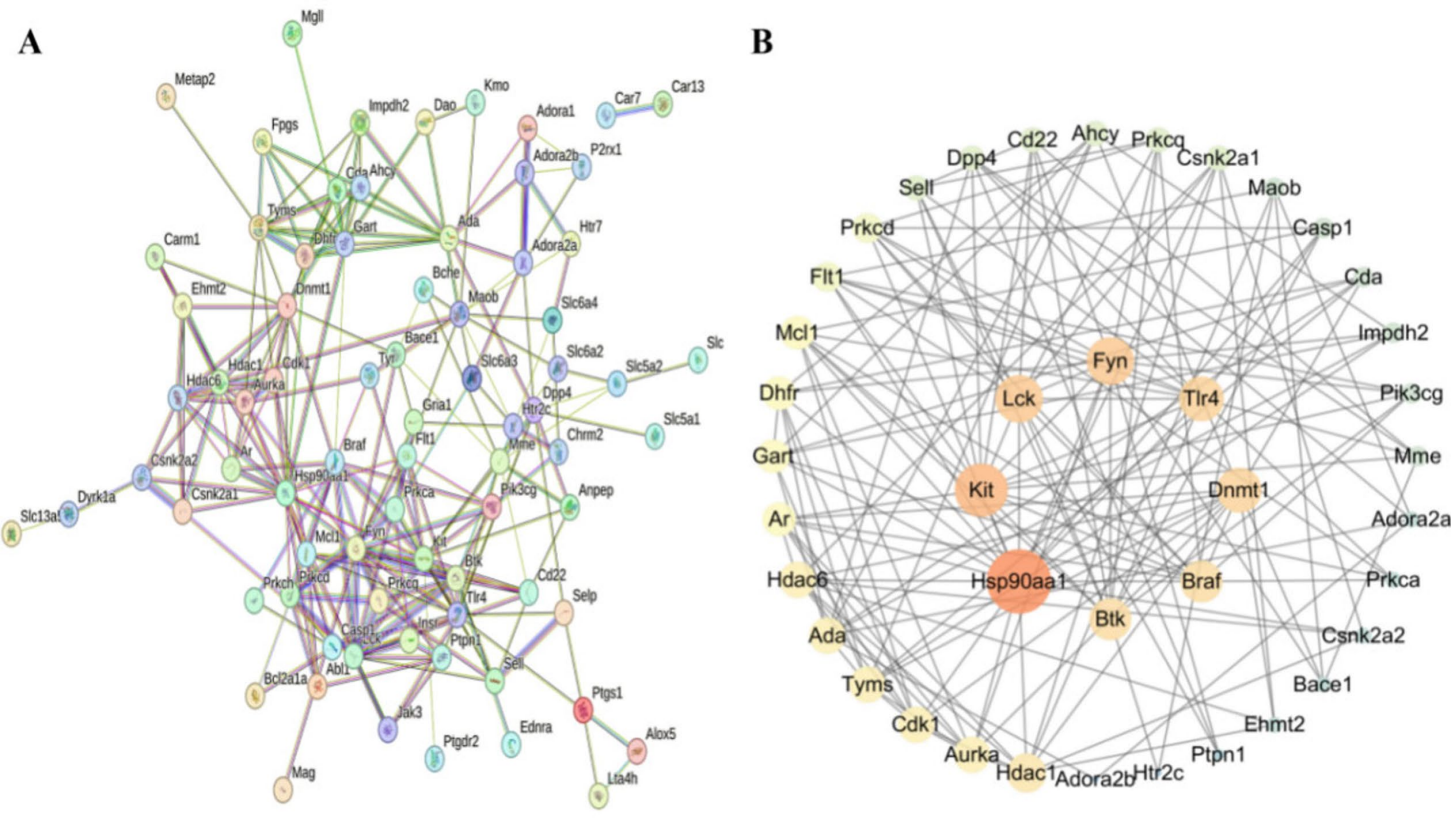
Results of CGA target protein network. **(A)** PPI network of potential targets of CGA. **(B)** PPI network diagram of potential core targets of CGA.

After inputting the 81 intersection target points into the STRING database, we obtained a total of 232 edges and 76 nodes. We calculated the average degree of each node to be 6.105 and determined the average local clustering coefficient to be 0.407 (Figure 3A).

To identify key target sites and analyze their interaction with body target sites, we imported information about the intersection target sites for CGA-oxidative stress into Cytoscape 3.8.0 software. This process allowed us to construct a network map for CGA-oxidative stress target sites (Figure 3B and Supplementary Table S1). Using the Analyze Network tool, we examined the degree values of each node. Hsp90aa1, Kit, Fyn, Lck, Tlr4, Maob, Dnmt1, Braf, Btk, and Mcl1 were the top 10 nodes. These results suggest that these target sites hold significant positions in the PPI network and are key targets for the action of CGA.

### 3.4 KEGG pathway enrichment results

**Figure 4:**
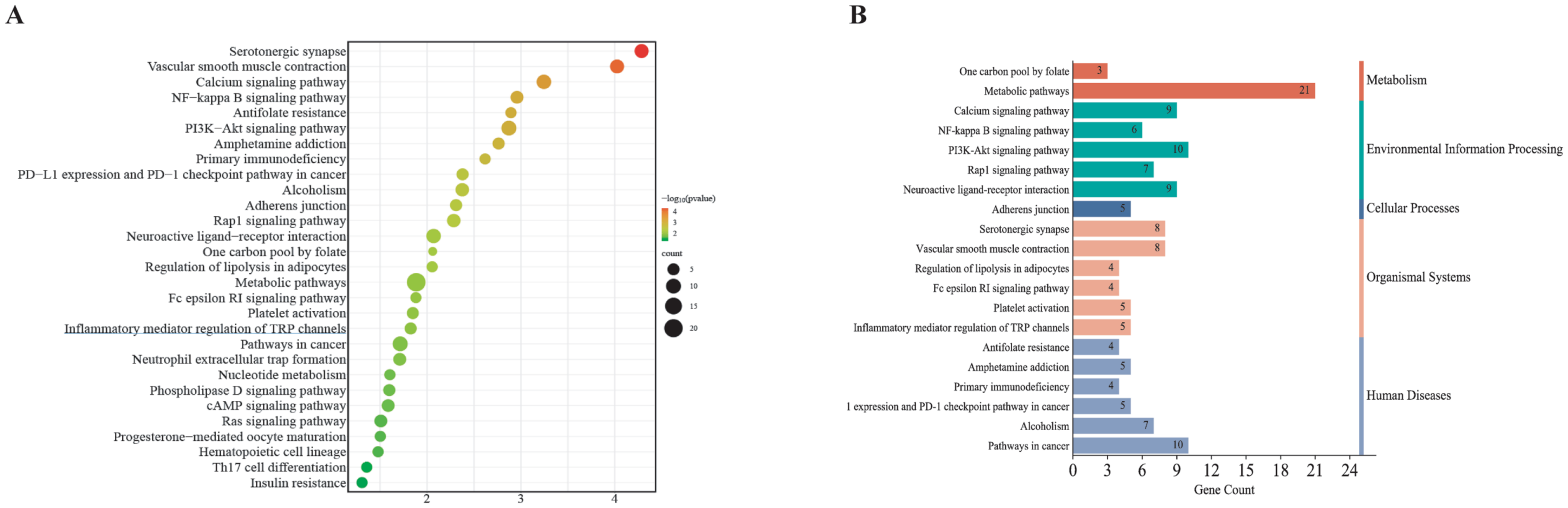
KEGG pathway analysis results. **(A)** KEGG enrichment bubble map. **(B)** KEGG enrichment bar chart.

**Figure 5:**
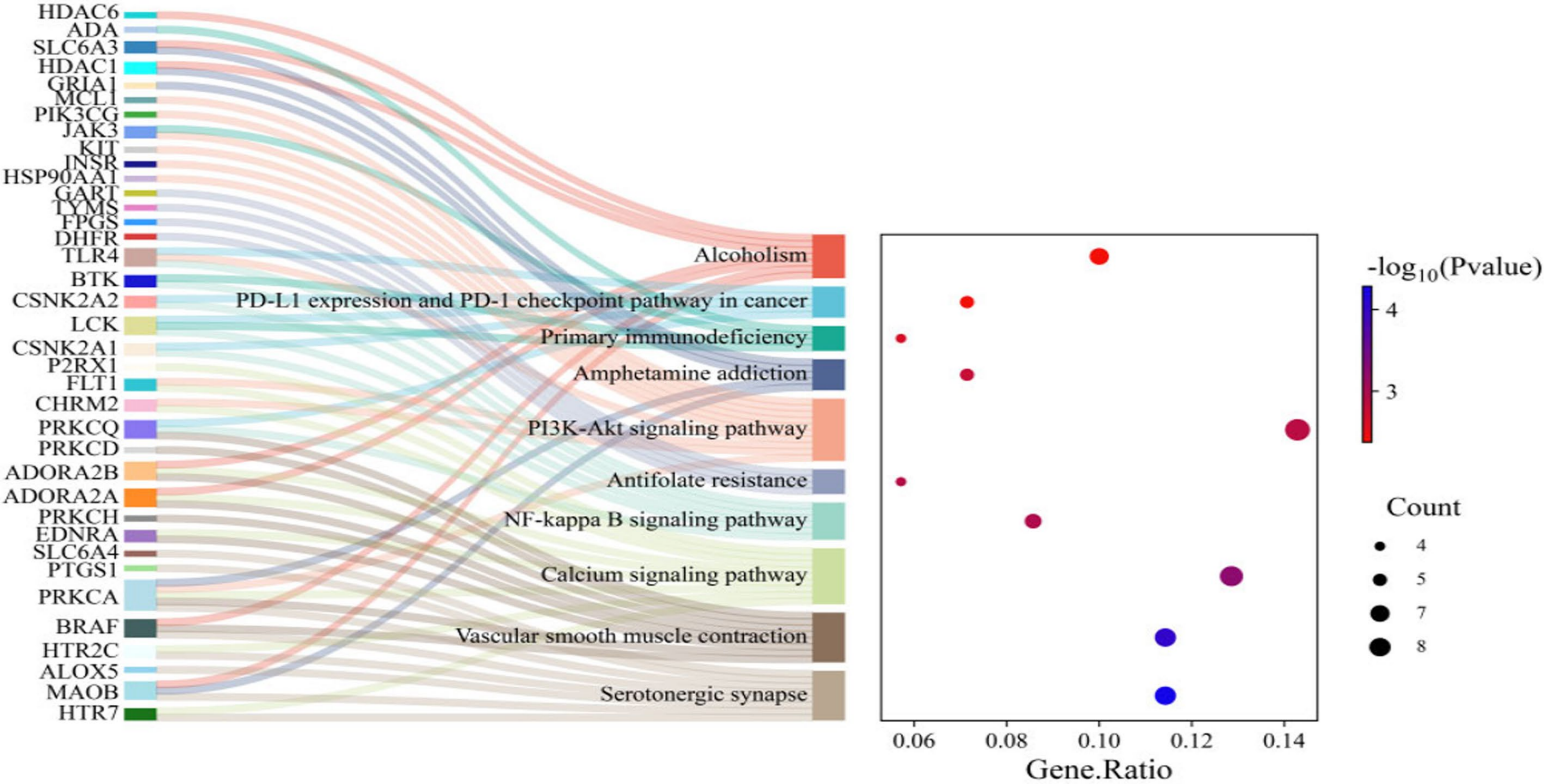
KEGG enrichment Sankey diagram.

KEGG signaling pathway enrichment analysis identified 39 signaling pathways. The key pathways associated with the antioxidant effects of CGA included vascular smooth muscle contraction, the calcium signaling pathway, the NF-κB signaling pathway, the PI3K/Akt signaling pathway, metabolic pathways, and various cancer-related pathways (Figure 4A and Supplementary Table S2). Figures 4B, 5 further highlight the PI3K/Akt signaling pathway as a central mechanism in CGA’s role in resisting oxidative stress.

### 3.5 Effect of CGA on MODE-K cells

**Figure 6:**
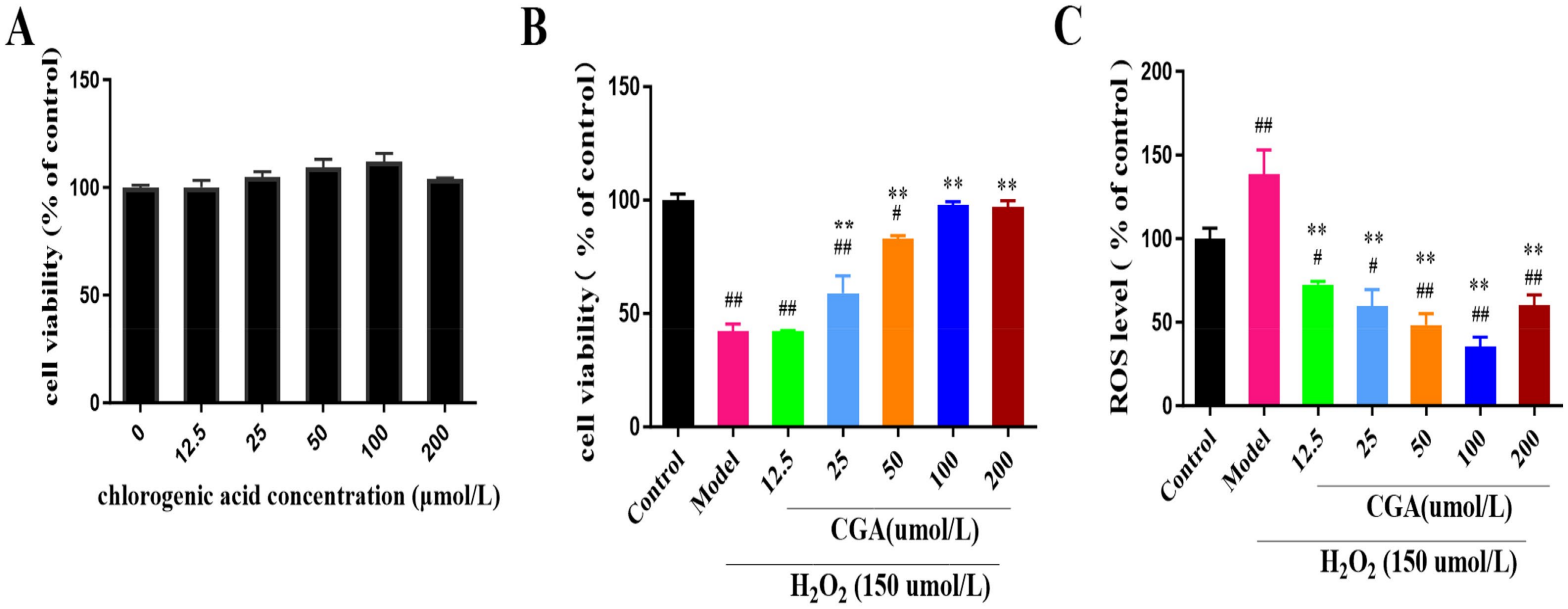
Effects of CGA on MODE-K intestinal epithelial cells. **(A)** Cytotoxicity of different concentrations of CGA in MODE-K cells; **(B)** Cell viability of MODE-K cells following CGA treatment; **(C)** Intracellular ROS levels in MODE-K cells treated with CGA. #*p* < 0.05, ##*p* < 0.01 vs Control; **p < 0.01 vs Model.

Figure 6A shows cell viability 12 h after treatment with CGA. The cell activity of the CGA group increased with increasing CGA concentration, unlike the effect noted in the blank control group. We noted a slight uptick in cell viability for CGA concentrations ranging from 12.5–100.0 μmol/L; however, CGA showed no significant cytotoxicity. These findings confirm that CGA is not toxic to MODE-K cells.

This was followed by experiments with different concentrations of H2O2, through which we determined that 150 μmol/L of H₂O₂ should be used for a duration of 4 h (Supplementary Table S3). Figure 6B shows that the cell survival rate in the damaged group significantly decreased to less than 50%. Conversely, the cell survival rate increased consistently with decreasing concentrations of CGA. Mouse small intestinal epithelial cells treated with 100 μmol/L CGA exhibited the highest survival rate, demonstrating a noteworthy enhancement of 55.86% compared with the cells of the animals in the damaged group. CGA pretreatment exhibited a time-dependent protective effect, with both 24 and 48 h treatments effectively maintaining cell viability (Supplementary Table S4). This finding suggests that CGA confers a protective impact against H₂O₂-induced cell damage and can efficaciously reverse oxidative stress damage induced by H₂O₂.

Figure 6C illustrates a rapid increase in the accumulation of ROS in cells treated with H₂O₂, with the ROS content in these cells significantly exceeding that of the cells in the untreated control group (*p* < 0.01). However, when compared with the injury group, ROS levels were reduced in a concentration-dependent manner following treatment with varying concentrations of CGA; ROS notably decreased after treatment with 100 μmol/L CGA (*p* < 0.01).

### 3.6 The effect of CGA on the accumulation of ROS within MODE-K cells induced by H₂O₂

**Figure 7:**
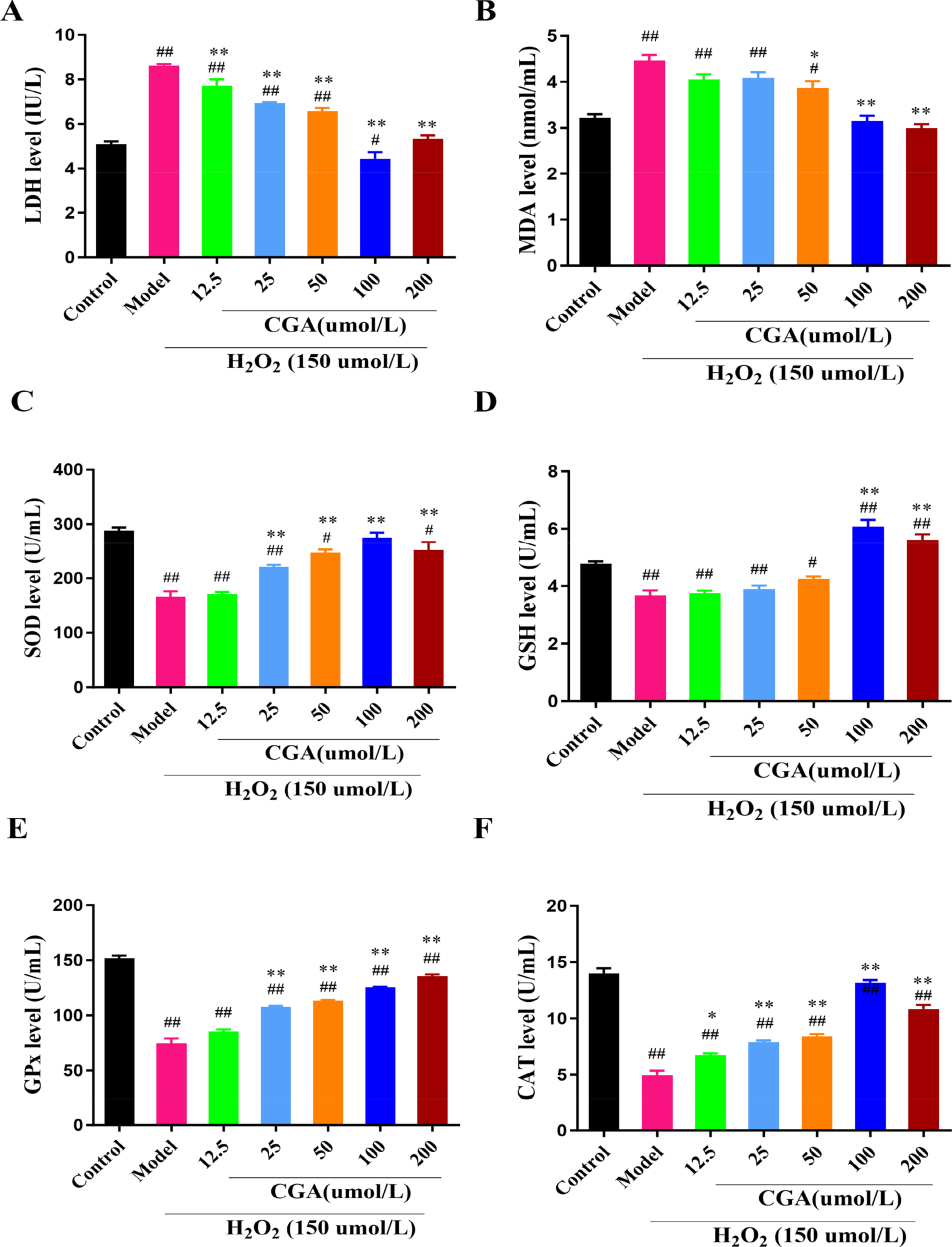
Effects of CGA on antioxidant indices of small intestinal epithelial cells in mice with oxidative stress. **(A)** Effects of CGA on LDH content and antioxidant enzyme activity in cells; **(B)** Effects of CGA on the MDA content in MODE-K cells; **(C)** Effects of CGA on the antioxidant enzyme activities content in MODE-K cells; **(D)** Effects of CGA on the content and ratio of GSH in MODE-K cells; **(E)** Effects of CGA on the content and ratio of GPx in MODE-K cells; **(F)** Effects of CGA on the content and ratio of CAT in MODE-K cells. #*p* < 0.05, ##*p* < 0.01 vs Control; *p < 0.05, **p < 0.01 vs Model.

Increasing concentrations of lipid peroxides can damage cell membranes. The release rate of LDH is viewed as an essential indicator of cell membrane integrity. Compared with the cells in the control group, the LDH content in the cells in the damaged group noticeably increased (*p* < 0.01); the LDH content in the CGA treatment group markedly decreased (*p* < 0.01) (Figure 7A; The symbols (#, *, &) were utilized to denote different levels of significance when comparing against distinct control groups). Compared with the damaged group, the LDH production prompted by H₂O₂ in the MODE-K cells, treated with either 100 μmol/L or 200 μmol/L CGA and H₂O₂, was significantly reduced (*p* < 0.01). These findings show that CGA can suppress lipid peroxidation and increase cell membrane permeability triggered by H₂O₂ in MODE-K cells. Among the treatments, the protective effect of 100 μmol/L CGA on cells was the most favorable. Therefore, we focused on this concentration in subsequent experiments.

The MDA content was significantly higher in the H₂O₂-induced damage group compared with the blank control group (*p* < 0.01), while the SOD level was notably decreased (*p* < 0.01) (Figures 7B,C). Nevertheless, we observed an increase in antioxidant enzyme activity correlated with increasing concentration of CGA; this trend was not noted in the model group. Simultaneously, the MDA content consistently decreased with the content of CGA increases. After the intervention of 100 μmol/L CGA, the lipid peroxide MDA levels appreciably decreased, and an increase in antioxidant SOD level was noted (*p* < 0.01). These findings suggest an enhancement in intracellular antioxidant levels post-CGA intervention.

After pretreatment with various concentrations of CGA, the activity of GSH in MODE-K cells exposed to H₂O₂ was evaluated. Figure 7D shows that GSH activity was significantly suppressed in the H₂O₂-treated group compared with the control group (*p* < 0.01). Conversely, we noted a significant increase in GSH activity in the groups treated with 100 μmol/L or 200 μmol/L CGA compared with the injury group (*p* < 0.01). The group treated with the 100 μmol/L concentration appeared to receive the most beneficial effect.

After pretreatment with various concentrations of CGA, we evaluated the activities of GPx and CAT in MODE-K cells exposed to H₂O₂. Figures 7E,F show that the activities of GPx and CAT in the H₂O₂-treated group were significantly lower than those in the control group (*p* < 0.01) [The symbols (#, *, &) were utilized to denote different levels of significance when comparing against distinct control groups]. In contrast, GPx activity was significantly increased in the CGA-treated group compared with the injured group (*p* < 0.01). The most significant effect of CAT enzyme activity was observed when the concentration of 100 μmol/L was used.

### 3.7 The effect of CGA on the apoptosis rate of cells

**Figure 8:**
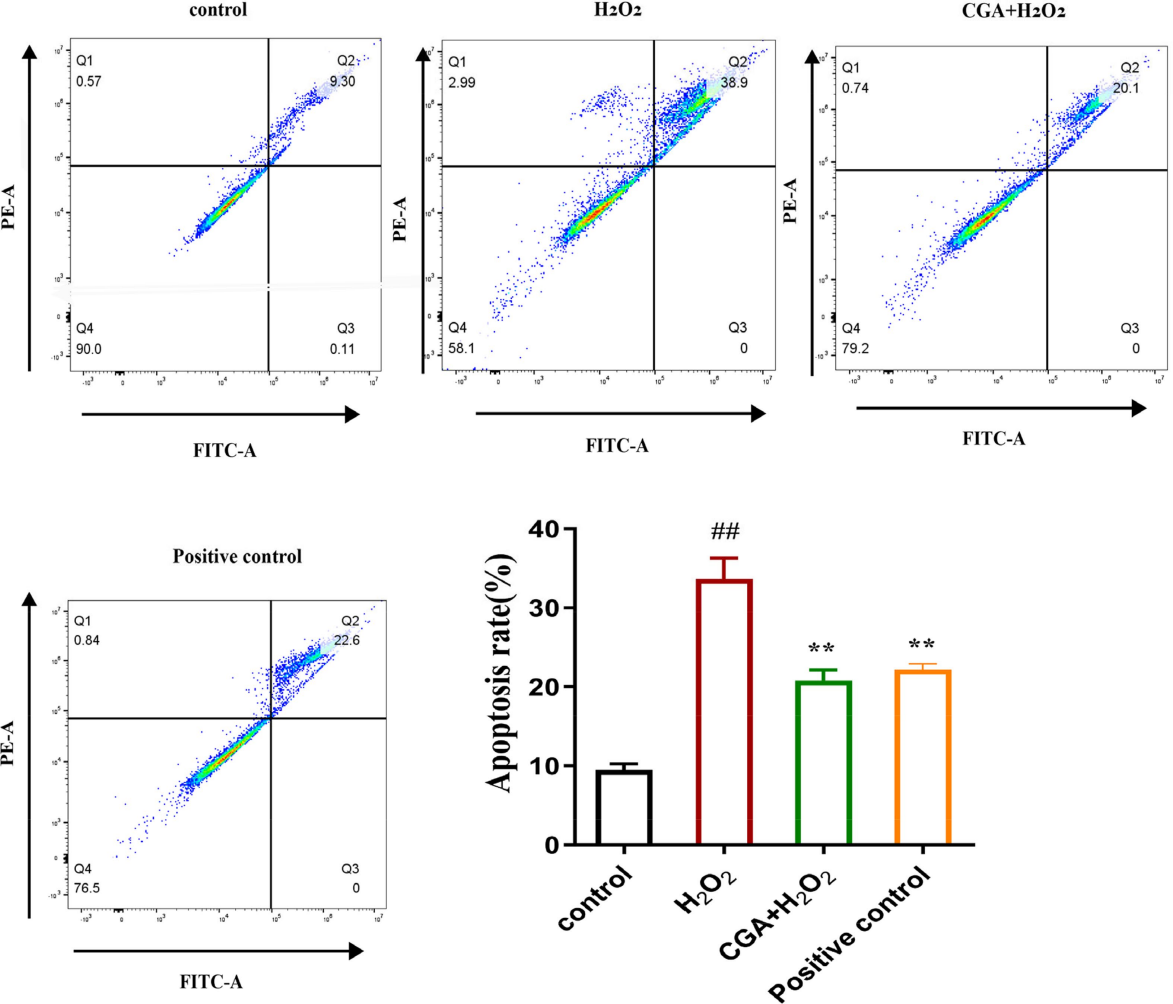
Effect of CGA on H_2_O_2_-induced apoptosis of small intestinal epithelial cells. ##*p* < 0.01 vs Control; **p < 0.01 vs H_2_O_2_.

We used the Annexin-V and propidium iodide double-staining technique. The Q4 region represents normal cells, the Q3 region signifies early apoptotic cells, the Q2 region indicates late apoptotic cells, and the Q1 region illustrates dead cells (Figure 8). The experimental procedure also resulted in mechanical cell death, as evident in the Q1 quadrant of the figure. Flow cytometry analysis revealed that the number of apoptotic cells in the injury group after H₂O₂ treatment was notably larger than that of the control group (*p* < 0.01). However, the number of apoptotic cells in the injury group significantly decreased compared with the untreated injury group with the CGA intervention (*p* < 0.01). The positive control group had the same effect as the CGA group after treatment.

### 3.8 The influence of CGA on apoptosis-related proteins

**Figure 9:**
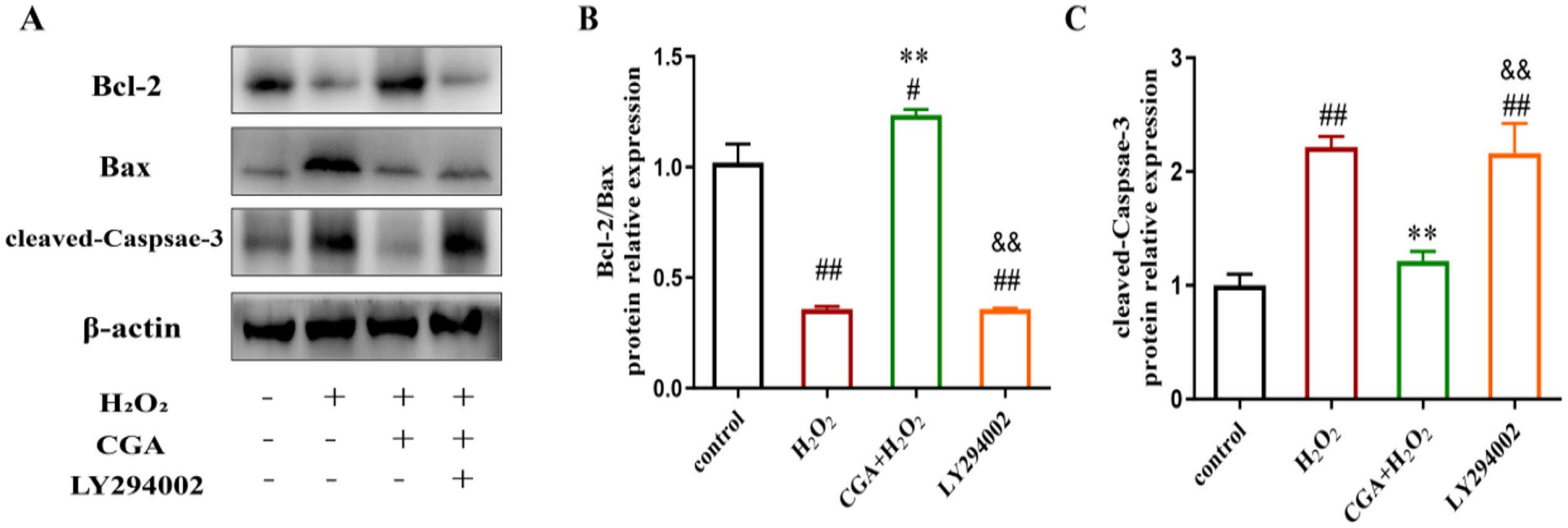
Impact of CGA on the expression of proteins in oxidative damaged cells induced by H₂O₂. **(A)** Western blot analysis of protein expression of Bcl-2/Bax; **(B)** The ratio of Bcl-2 to Bax is quantified to reflect the anti-apoptotic ability of the cells; **(C)** protein expression of cleaved-Caspase-3. #*p* < 0.05, ##*p* < 0.01 vs control; **p < 0.01 vs H_2_O_2_; &&*p* < 0.01 vs CGA+H_2_O_2_.

Compared with the blank control group, the protein levels of Bcl-2/Bax in the H₂O₂ treatment group were significantly decreased (*p* < 0.01). The protein levels of Bcl-2/Bax in the CGA intervention group were significantly increased (*p* < 0.01), and the protein level of cleaved-Caspase-3 was significantly decreased *p* < 0.01). The protein level of cleaved-Caspase-3 in the LY294002 inhibition group was significantly increased (*p* < 0.01). CGA alleviated the oxidative damage of H₂O₂ to MODE-K cells by up-regulating the expression of Bcl-2 in the Bcl-2/Bax/Caspase-3 signaling pathway and down-regulating the protein levels of Bax and Caspase-3 (Figures 9A,B,C).

### 3.9 CGA and Keap1 molecular docking diagram

**Figure 10:**
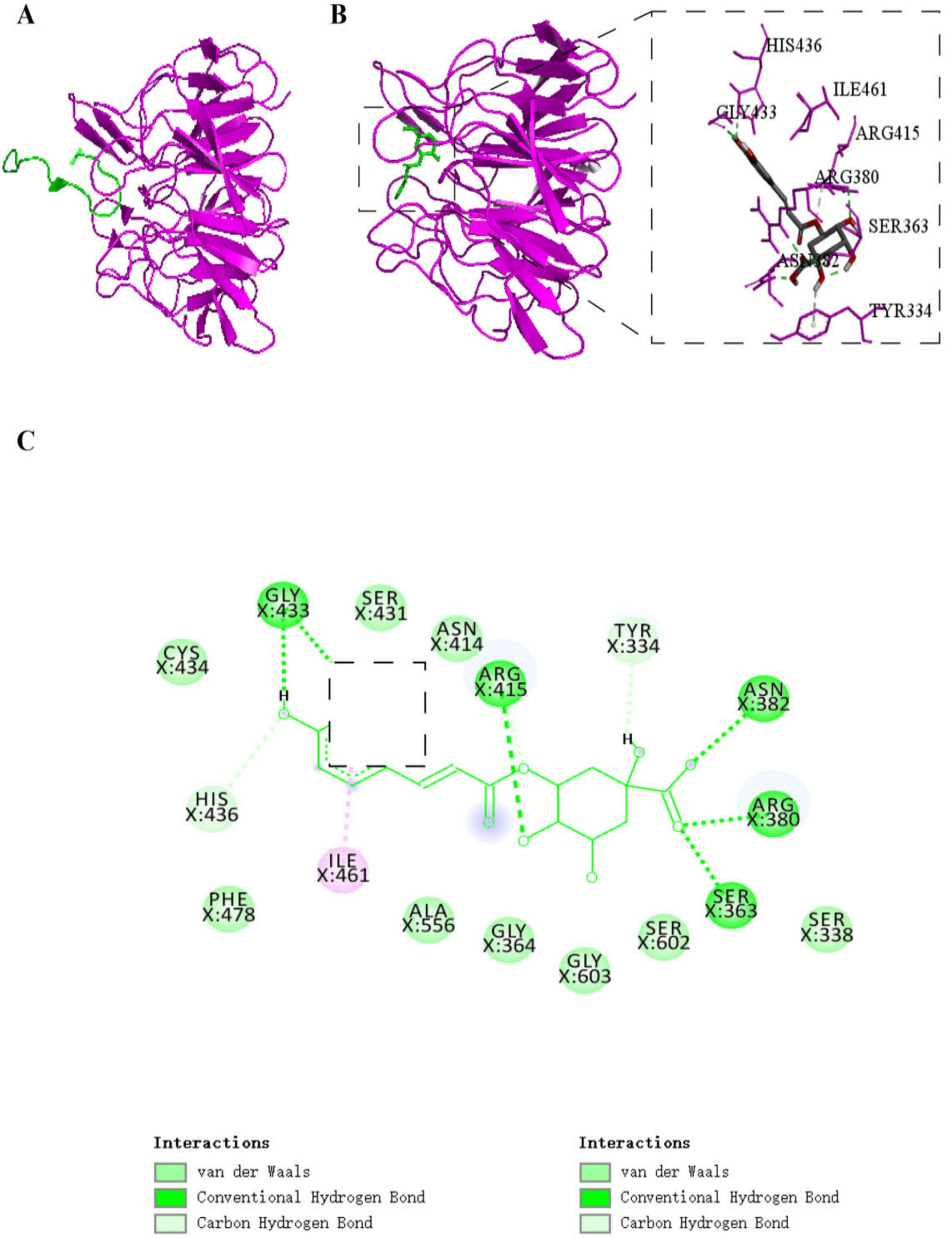
Model of CGA interacting with Keap1. **(A)** 3D diagram of Keap1 and Nrf2 complex; **(B)** 3D diagram of CGA and Keap1 complex; **(C)** Two-dimensional diagram of the interaction between CGA and Keap1.

The preferential conformation and interaction of CGA with the Keap1-Nrf2 complex can be understood via molecular simulation technology. This technology illustrates the mechanism by which CGA inhibits the interaction between Keap1 and Nrf2. Figures 10A,B show the three-dimensional structures of the Nrf2 and the Kelch region of Keap1, also known as the Keap1 pocket. We showed that CGA binds to the Kelch binding region similarly to the Nrf2-Keap1 interaction. Molecular docking showed that CGA binds to Keap1 Kelch domain with high affinity (binding energy: −8.96 kcal/mol). Figure 10C shows the CGA-Keap1 pocket binding interaction model. Here, CGA interacts with the Keap1 pocket via hydrogen bonds, and the associated amino acid residues (Arg380, Arg415, Ser363) are integral to the Nrf2-Keap1 binding. This discovery suggests that CGA fills the Nrf2 binding site in the Keap1 Kelch, hindering the interaction between Keap1 and Nrf2 and thereby conferring antioxidant effects.

### 3.10 Detection of basic indicators and intestinal injury-related indicators in experimental mice *in vivo*

**Figure 11:**
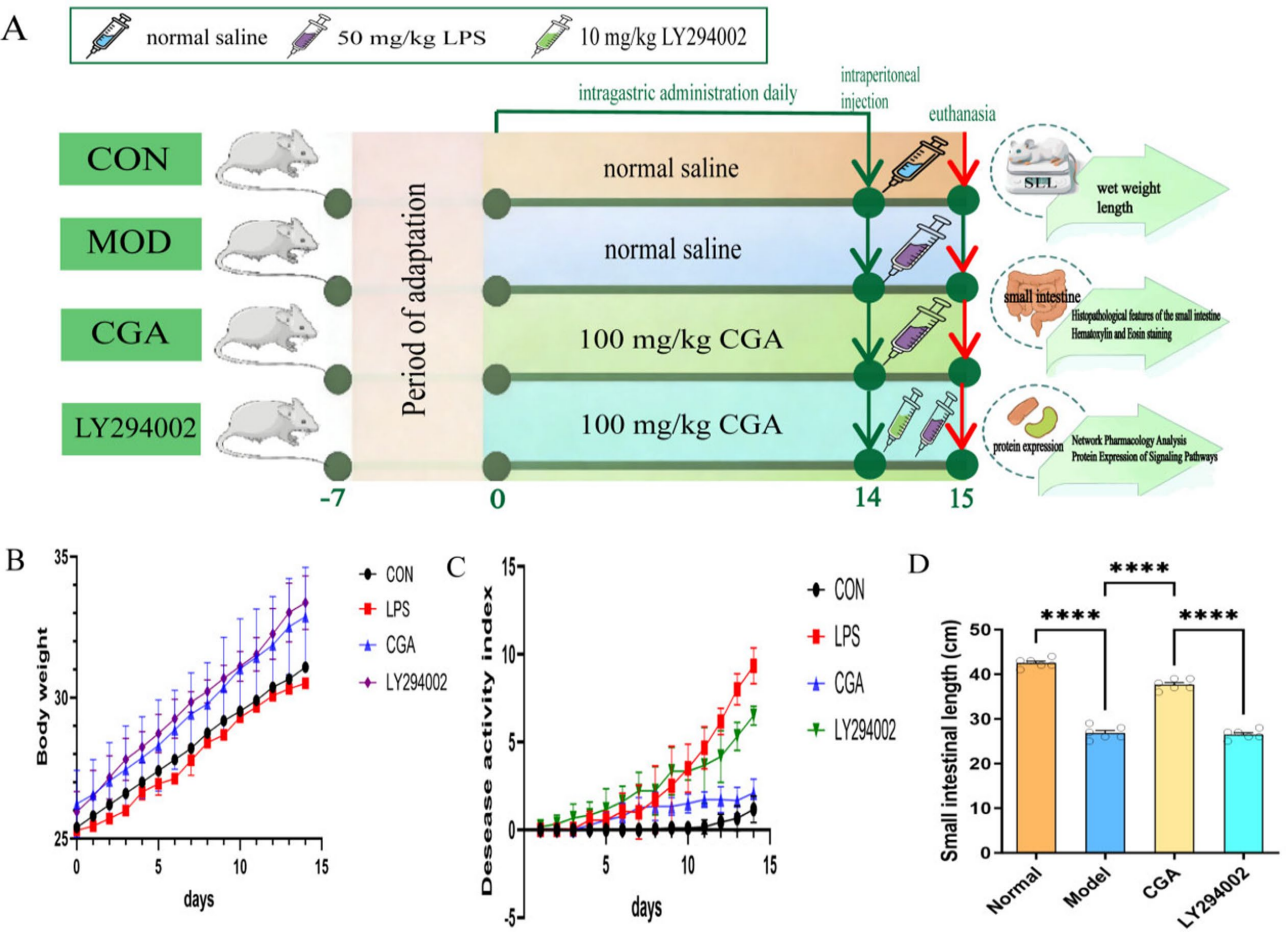
**(A)** Timeline and grouping diagram of animal experiments; **(B)** The line graph showing the changes in the growth weight of mice after LPS modeling and CGA intervention; **(C)** Analysis of the curve results of the mouse disease activity index (DAI); **(D)** Analysis of the bar chart results of the length of the mouse small intestine. ****p < 0.0001.

Figure 11A shows the grouping and timeline of the in vivo experiments in mice, clarifying the complete process of adaptation, gavage intervention, modeling, and sample collection, providing an experimental basis for subsequent results. Figure 11B indicates that LPS modeling significantly inhibits weight gain in mice; CGA intervention can significantly improve weight loss; and the PI3K inhibitor LY294002 can block the protective effect of CGA, suggesting that the growth-promoting effect of CGA depends on the PI3K/Akt pathway. Figure 11C shows that LPS modeling causes a continuous increase in the DAI score of mice and aggravates intestinal inflammation; CGA can significantly reduce DAI and alleviate intestinal inflammation; LY294002 can reverse the anti-inflammatory effect of CGA, verifying that the anti-inflammatory effect of CGA depends on the PI3K/Akt pathway. Figure 11D shows that LPS modeling leads to significant atrophy and shortening of the small intestine in mice; CGA intervention can effectively protect the intestinal structure and restore intestinal length; LY294002 can counteract the protective effect of CGA, once again confirming that the protection of CGA on the intestinal structure depends on the PI3K/Akt pathway.

### 3.11 CGA ameliorates LPS-induced intestinal tissue injury, inflammatory infiltration and apoptosis in mice

**Figure 12:**
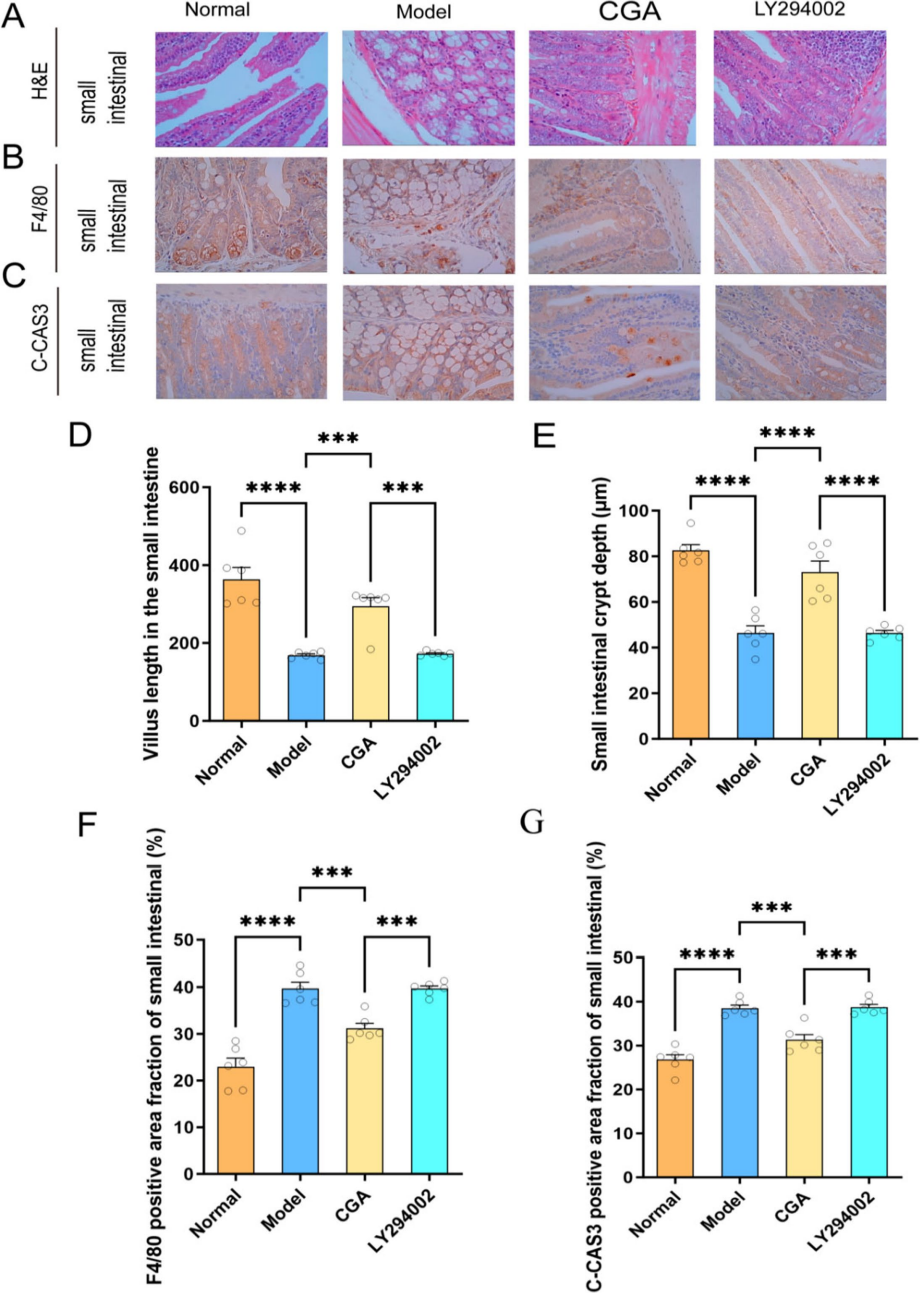
**(A)** Analysis of pathological results of H&E staining of small intestinal tissue; **(B)** Analysis of F4/80 immunohistochemical results of small intestinal tissue; **(C)** Analysis of immunohistochemical results of C-CAS3 in small intestinal tissue; **(D)** Analysis of quantitative results of small intestinal villus length; **(E)** Quantitative analysis of the depth of intestinal crypts; **(F)** Quantitative analysis of the positive area fraction of F4/80; **(G)** Quantitative analysis of the positive area fraction of C-CAS3. ***p < 0.001, ****p < 0.0001.

Figure 12A shows the pathological morphology of the small intestinal tissues of four groups of mice: the normal group has a complete structure; the model group (Model) shows obvious inflammation and villus atrophy; the CGA group shows a significant improvement in pathological damage; the LY294002 group (inhibitor) again shows severe damage, verifying that the protective effect of CGA depends on the PI3K/Akt pathway. Figure 12B indicates the small intestinal immunohistochemistry, F4/80 is a macrophage marker: the positive staining in the model group significantly increases (inflammation infiltration intensifies); the CGA group shows a significant decrease in positive expression (inflammation alleviates); the positive staining in the LY294002 group recovers, confirming that CGA can inhibit intestinal inflammation infiltration, and this effect is blocked by the PI3K inhibitor. Figure 12C represents the C-CAS3 immunohistochemistry, C-CAS3 is a cell apoptosis marker: the number of apoptotic cells in the model group significantly increases; the apoptotic level in the CGA group is significantly reduced; the apoptotic level in the LY294002 group increases again, indicating that CGA can inhibit LPS-induced intestinal cell apoptosis, and this effect depends on the PI3K/Akt pathway. Figure 12D shows the statistical results of small intestinal villus length, the villus length in the model group is significantly shortened; the villus length after CGA intervention is significantly restored; the villus length in the LY294002 group shortens again, quantitatively verifying the protective effect of CGA on the intestinal mucosal structure. Figure 12E shows the statistical results of small intestinal crypt depth, the crypt depth in the model group significantly becomes shallower; CGA can effectively restore the crypt depth; the crypt depth in the LY294002 group decreases again, further confirming the protective effect of CGA on the intestinal structure. Figure 12F shows the statistical results of the F4/80 positive area rate, through the quantitative Figure 12B: the F4/80 positive area rate in the model group significantly increases; the positive rate in the CGA group significantly decreases; the positive rate in the LY294002 group recovers, objectively verifying the anti-inflammatory effect of CGA. Figure 12G shows the statistical results of the C-CAS3 positive area rate, through the quantitative Figure 12C: the C-CAS3 positive area rate in the model group significantly increases; the positive rate in the CGA group significantly decreases; the positive rate in the LY294002 group recovers, objectively verifying the anti-apoptotic effect of CGA.

### 3.12 CGA regulates the expression of LPS-induced oxidative stress-related proteins in mouse small intestine through the PI3K/Akt-Nrf2/HO-1 pathway

**Figure 13:**
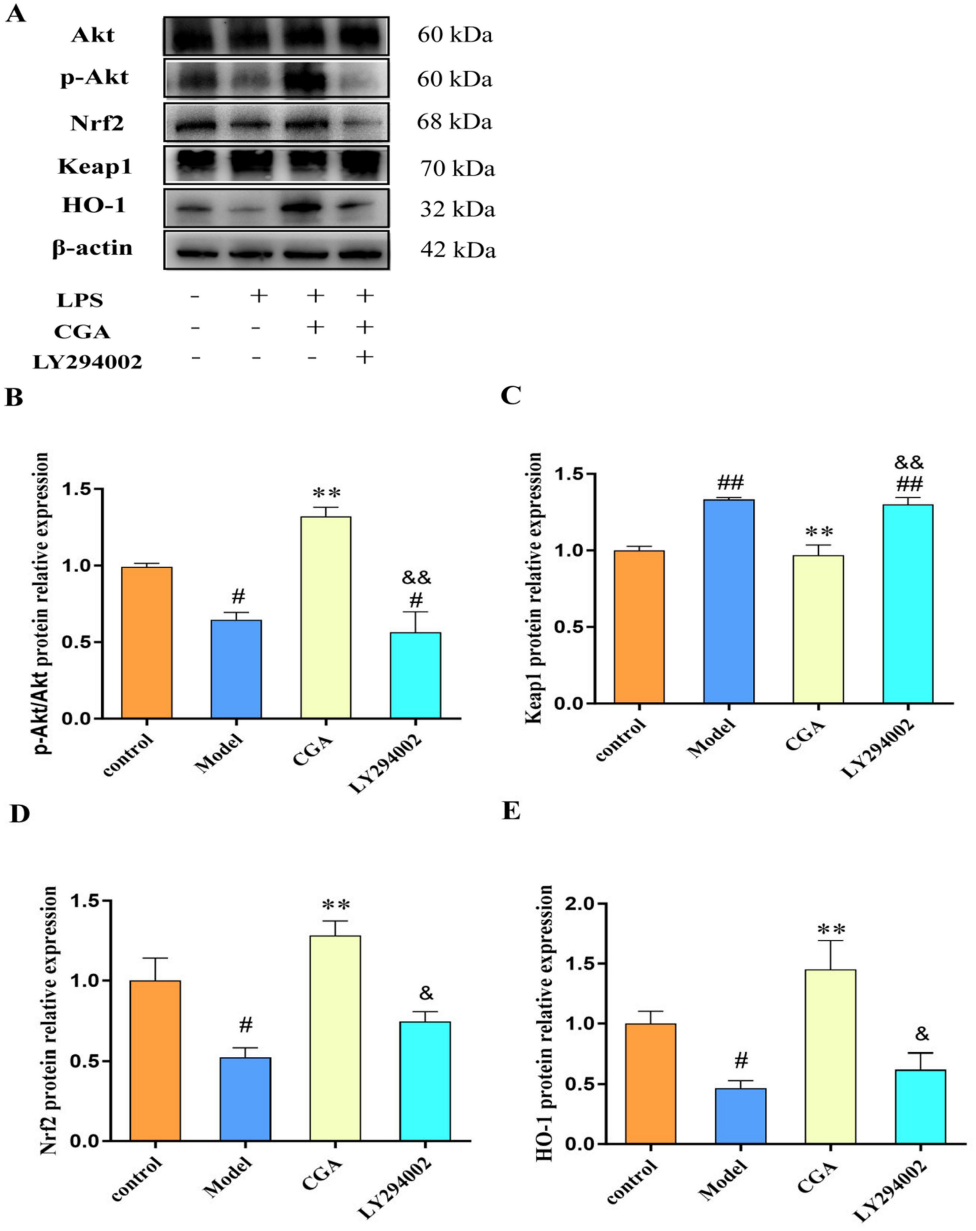
Effect of CGA on the p-Akt/Akt/Nrf2/HO-1 pathway-associated proteins in LPS-induced small intestinal tissue. **(A)** Western blot analysis of protein expression of p-Akt, Akt, Nrf2, Keap1, and HO-1; **(B)** protein expression of p-Akt/Akt; **(C)** protein expression of Keap1; **(D)** Nrf2 protein expression; **(E)** HO-1 protein expression. #*p* < 0.05, ##*p* < 0.01 vs control; **p < 0.01 vs Model; &*p* < 0.05, &&*p* < 0.01 vs CGA.

Figure 13A presents the original Western Blot banding diagram, showing the protein expression bands of Akt, p-Akt, Nrf2, Keap1, HO-1, and the internal reference *β*-actin in the small intestinal tissues of each group of mice. It visually presents the differences in protein levels under different interventions. Figure 13B represents the relative expression of p-Akt/Akt. The ratio of p-Akt/Akt in the model group (Model) was significantly decreased; the ratio in the CGA group was significantly increased; the LY294002 inhibitor group could reverse the effect of CGA, confirming that CGA can activate the PI3K/Akt pathway. Figure 13C shows the relative expression of Keap1. The expression of Keap1 in the model group was significantly increased; the expression in the CGA group was significantly decreased; the expression of Keap1 in the LY294002 group returned, indicating that CGA can inhibit Keap1, and this effect depends on the PI3K/Akt pathway. Figure 13D represents the relative expression of Nrf2. The expression of Nrf2 in the model group was significantly decreased; the expression in the CGA group was significantly increased; the expression of Nrf2 in the LY294002 group decreased, confirming that CGA can promote Nrf2 activation, dependent on the PI3K/Akt pathway. Figure 13E shows the relative expression of HO-1. The expression of HO-1 in the model group was significantly decreased; the expression in the CGA group was significantly increased; the expression of HO-1 in the LY294002 group decreased, verifying that CGA regulates the expression of HO-1 upstream through the PI3K/Akt pathway, exerting antioxidant effects.

## 4 Discussion

Oxidative stress refers to the physiological response induced in organisms when they are damaged by oxidative stress factors (36). At the cellular level, oxidative stress can cause oxidative damage to proteins, lipids, and nucleic acids, thereby affecting normal metabolic processes within cells and disrupting the nutritional metabolism balance and physiological homeostasis of the body. This phenomenon is closely related to various diseases such as cardiovascular diseases, enteritis, and colorectal cancer (37), and is also a key factor affecting the health of animals, the growth performance of livestock and poultry, and the efficiency of nutrient utilization. Therefore, it has become a key research object. Chlorogenic acid, as a natural polyphenolic compound, is also a functional nutritional factor that has attracted much attention in the fields of functional foods and animal nutrition, and has significant antioxidant activity (38). Song et al. studied the protective effect of chlorogenic acid on oxidative stress in lens epithelial cells induced by hydrogen peroxide and found that it could significantly reduce the apoptosis rate of cells under oxidative stress conditions and enhance the expression of antioxidant response-related proteins (39). The Gao team found that chlorogenic acid could reduce the apoptosis rate of cells treated with oxidative stress and enhance cell viability by regulating the autophagy process, thereby protecting cells from oxidative damage (40). However, the specific mechanism by which chlorogenic acid protects cells from oxidative damage remains unclear, especially its targeted action mechanism in animal nutrition regulation and functional food development, which still needs to be further explored. This study used network pharmacology to analyze the potential mechanism of chlorogenic acid in antioxidative stress, providing theoretical support for its application in functional food research and development and animal nutrition regulation. Network pharmacology, as a new cost-effective research method integrating knowledge from bioinformatics, systems biology, and pharmacology, has broken through the traditional “single drug–single target” model and constructed a new paradigm for drug research and development based on the principles of network and systems biology. It also provides an efficient research tool for the mechanism analysis of functional nutrients. This technology is gradually revealing the unknown mechanisms of action of more and more Chinese herbal medicines and their components (41), providing new ideas for the development of functional foods and animal nutrition additives. When Wang’s team used network pharmacology to study the effect of chlorogenic acid against COVID-19, they found 146 GO terms related to various biological functions, molecular functions, and cellular components through GO enrichment analysis, and KEGG analysis showed that 70 protein targets were associated with 46 pathways, suggesting that chlorogenic acid may treat COVID-19 through pathway regulation (42). Zhou et al. found that chlorogenic acid was associated with the down-regulation of the SRC/MAPKs signaling pathway when exploring its potential therapeutic targets for glioma (43). When Xia’s team evaluated the efficacy and mechanism of action of Erbao Pill in treating periodontitis, they pointed out through GO and KEGG analysis that the key components berberine and chlorogenic acid acted on key target genes by regulating the PI3K/Akt and NF-κB/MAPK signaling pathways (44). Chlorogenic acid mainly achieves antioxidant effects by influencing biological processes such as protein phosphorylation and responding to external stimuli, involving 39 antioxidant stress response pathways including PI3K/Akt, NF-κB, and calcium ions, demonstrating its potential application value as an antioxidant stress drug, a functional food factor, and an animal nutrition additive. We have observed that in many studies within the same field that do not focus on the pathway mechanism, they often use the intervention of upstream and downstream protein expression combined with inhibitors to support the activation of the signaling axis. They did not conduct detection of Nrf2 nuclear translocation, which is a relatively common research paradigm in this field (45).

Biological functions encompass a wide range of physiological and pathological processes, including oxidative stress damage. These processes are regulated by multiple signaling pathways, among which the PI3K/Akt pathway is one of them (46). As a functional food factor and an active component in animal nutrition, the study of the targeted mechanism of chlorogenic acid (CGA) is of great significance for improving animal health and nutritional utilization efficiency. Gong and his team evaluated the protective effect of *Eucommia ulmoides* leaf extract on gastric ulcers and explored its potential mechanism. They found that the extract contains neochlorogenic acid, chlorogenic acid (CGA), and rutin, which may alleviate oxidative stress and inflammatory responses by regulating the PI3K/Akt/NF-κB signaling pathway (47). In another study, Chen et al. used hydrogen peroxide (H₂O₂) to treat human umbilical vein endothelial cells to establish an oxidative stress injury model, and then pretreated these cells with a lignan flavonoid. They found that this substance could effectively resist H₂O₂-induced oxidative damage through the PI3K/Akt pathway (48). This study used network pharmacology technology to hypothesize that CGA may protect the body from H₂O₂-induced oxidative stress damage by activating the PI3K/Akt pathway. To verify this computational prediction result, we will conduct *in vitro* and/or *in vivo* animal experiments in subsequent studies to provide more direct scientific evidence for its application in animal nutrition regulation and functional feed development. Oxidative stress results from an imbalance in the body’s redox state, which has a significant impact on animal health (49, 50). In animal nutrition and functional food research, the body’s antioxidant capacity is often used as a core indicator to evaluate the effect of nutritional intervention. Glutathione (GSH) can eliminate organic hydrogen peroxides and H₂O₂, two key antioxidant enzymes, while malondialdehyde (MDA) is often used as an indicator to measure the degree of lipid peroxidation in mammals (51). Therefore, the serum levels of these markers are often used to monitor the body’s antioxidant capacity. Our research shows that as a potential functional nutritional additive, supplementing CGA can alleviate H₂O₂-induced small intestinal oxidative damage in mice by increasing GSH activity and reducing MDA levels. In MODE-K cells, chlorogenic acid enhances the antioxidant capacity of cells by increasing the activity of antioxidant enzymes. Similarly, Shang et al. found that adding CGA to the feed could significantly increase the activities of superoxide dismutase (SOD) and GSH and reduce MDA content, not only improving the growth performance, immune function, and antioxidant capacity of common carp, but also promoting the health of their intestines and livers (52). The research team of Zha investigated the protective effect of CGA on oxidative stress induced by paraquat in broilers and found that the total antioxidant capacity, catalase, superoxide dismutase, and glutathione peroxidase activities in the serum and liver of the treatment group were all increased, while MDA accumulation was reduced. This indicates that CGA may improve the growth performance of broilers affected by paraquat, alleviate oxidative stress, and reduce liver inflammation (53). Gu et al. pointed out that CGA can protect cells from lipopolysaccharide (LPS)-induced cell death and reduce the generation of oxygen free radicals. After treatment with CGA, the activity of cellular antioxidant enzymes was significantly increased, with particularly significant increases in SOD and GSH activities, which has positive implications for regulating inflammatory diseases (54). These findings are highly consistent with our research results that CGA protects MODE-K cells from oxidative stress by enhancing cellular antioxidant capacity, and also provide strong support for the application of CGA in functional foods and animal nutritional additives.

The expression of multiple key antioxidant genes also enhanced the improvement of antioxidant capacity. Nrf2, as the main regulatory factor of the antioxidant response, helps to regulate the expression levels of innate antioxidant enzymes, which can resist oxidative stress and maintain the redox balance of the organism, which is crucial for the nutritional metabolism and health homeostasis of the animal body (55). Chen et al. reported that by increasing the mRNA expression level of Nrf2, the activity of antioxidant enzymes can be enhanced (56). During the treatment of mouse epithelial cells, chlorogenic acid (CGA) activated the Nrf2 signaling pathway and significantly accelerated the migration of Nrf2 to the nucleus. This faster migration, in turn, made the gene expression regulation more timely and effective, protecting cells from damage caused by oxidative stress. El-Khadragy et al. studied the protective effect of CGA on testicular dysfunction induced by sodium arsenite, and found that pretreatment with CGA in mice could significantly improve the testicular damage caused by arsenic, and its mechanism may be related to the Nrf2 signaling pathway. With its antioxidant, anti-inflammatory and anti-apoptotic properties, CGA can alleviate the reproductive toxicity caused by arsenic (57), highlighting its potential application in animal health maintenance as a functional nutrient component. Liu et al. found that CGA has a significant protective effect on the growth performance and intestinal health of broilers under the influence of dexamethasone. Adding CGA as a functional nutritional additive to the feed can activate the Nrf2 pathway regulated by autophagy, enhance the antioxidant capacity of the organism and reduce the level of cell apoptosis. Moreover, compared with non-stress conditions, the addition of CGA can improve the stress resistance of broilers and improve the production performance of livestock (58). Chen et al. studied the protective effect of CGA on intestinal epithelial cells under oxidative stress and the potential mechanism, indicating that CGA can alleviate the intestinal inflammation and damage caused by oxidative stress in weaned piglets, which is of great reference value for solving the problem of stress-induced injury in piglets in livestock production and optimizing animal nutrition regulation strategies. Chlorogenic acid can phosphorylate two key signaling proteins Akt and Nrf2 in the PI3K/Akt pathway, thereby promoting the increase in the expression of antioxidant enzymes and heme oxygenase-1 in the cell. Therefore, CGA may alleviate the inflammatory and damage of intestinal epithelial cells caused by oxidative stress through regulating the PI3K/Akt signaling pathway (59). Given its antioxidant and anti-inflammatory properties, CGA has become a potential candidate substance for preventing or treating various oxidative stress injuries and diseases, and also has broad application prospects in functional food research and the development of green nutritional additives for livestock breeding.

Apoptosis is often associated with oxidative stress. When the level of reactive oxygen species (ROS) inside the cells is too high, biological macromolecules such as DNA, proteins, and lipids may be damaged and trigger apoptosis. As a functional food factor and an active component of animal nutrition, chlorogenic acid (CGA) has the ability to regulate cell apoptosis, which is of great significance for maintaining the health of the animal body and optimizing nutritional intervention plans. Numerous studies have shown that CGA can eliminate excessive ROS induced by chemical substances, alleviate oxidative stress-related cellular damage, and inhibit cell apoptosis. Moslehi et al. studied the protective effect of CGA on endoplasmic reticulum stress-induced inflammation and apoptosis in mice induced by streptomycin, and found that CGA reduced the expression of key inflammatory and apoptotic factors NF-κB and Caspase-3, improving the state of hepatocyte apoptosis and inflammation (60). The Zada team investigated the mechanism by which CGA protects human chondrocytes from apoptosis induced by H₂O₂, confirming that CGA treatment combined with autophagy induction can significantly reduce apoptosis caused by ROS (61). Dkhil et al. examined the toxicity of sodium arsenite on mice and found that CGA could reverse the biochemical, molecular, and histological changes caused by sodium arsenite, demonstrating its liver-protective function. They proposed that CGA could block the apoptosis triggered by NaAsO₂ by down-regulating Bax and Caspase-3 and up-regulating Bcl-2, indicating that CGA has the potential to become a nutritional supplement for treating liver damage caused by sodium arsenite (62). This study found that CGA not only enhances the antioxidant capacity of cells but also inhibits apoptosis by regulating the Bcl-2/Bax/Caspase3 signaling pathway, which is consistent with the aforementioned studies and provides strong support for its application in animal nutrition regulation and functional feed development. To explore the interaction between small molecules and target proteins, molecular docking technology has been widely used. This technology has important application value in the interpretation of the mechanism of functional nutritional components, the development of functional foods, and the screening of animal nutrition additives. Xiong et al. studied the potential targets and molecular mechanisms of CGA in treating neuroinflammation. Through network pharmacology prediction combined with molecular docking verification, they determined that the TNF signaling pathway was the key pathway for CGA to exert anti-neuroinflammatory effects, and Akt1, TNF, MMP9, PTGS2, MAPK1, MAPK14, and RELA were identified as the core targets of CGA (63). Velázquez-Enríquez’s team used molecular docking to precisely locate potential therapeutic targets when studying the molecular mechanism of CGA in treating idiopathic pulmonary fibrosis, confirming that CGA could stably bind to the target protein (64). The conclusion of this study is highly consistent with the discovery of our team: CGA could disrupt the binding site of Keap1 protein’s Kelch domain and Nrf2, thereby interfering with the Keap1-Nrf2 interaction. Molecular docking analysis suggests a potential interaction between CGA and the Kelch domain of Keap1, which may interfere with the Keap1-Nrf2 protein–protein interaction. This hypothesis-generating finding provides a plausible mechanism for Nrf2 release and warrants future biochemical validation. This process regulates the expression of Nrf2 and HO-1 to improve the antioxidant state of intestinal cells, effectively preventing H₂O₂-induced damage to intestinal cells, and ultimately inhibiting apoptosis while demonstrating antioxidant effects. This mechanism not only provides a scientific basis for CGA as an antioxidant nutrient but also provides theoretical support for its application in functional food development and livestock and poultry health breeding.

This study demonstrates that chlorogenic acid (CGA) exhibits a unique dual mechanism of action compared to traditional antioxidants such as vitamin C. While directly eliminating free radicals, CGA can also activate cellular defense pathways - including the Keap1-Nrf2 interaction and the PI3K/Akt signaling pathway, thereby more effectively regulating chronic oxidative stress. As a highly promising functional nutritional component and novel animal nutrition additive, CGA’s dual mechanism of action gives it unique advantages in the development of functional foods and the healthy breeding of livestock. Although CGA shows good safety at physiological concentrations, comprehensive toxicological studies in the veterinary field are still necessary (65). Although *in vitro* experiments can precisely analyze the mechanism of action, they cannot fully simulate the complex physiological environment of the entire organism (66). It is notable that we found that CGA has a direct local effect on intestinal epithelium before systemic absorption (67), which supports its potential as a dietary supplement and functional feed additive for treating intestinal diseases in livestock under stress conditions, potentially reducing the use of traditional antibiotics (68). Further *in vivo* experiments are needed to verify the physiological relevance of these mechanism discoveries and to assess the efficacy and safety of CGA at the overall animal level.

Oxidative stress and inflammatory response are key links in LPS-induced intestinal damage, leading to the destruction and dysfunction of the intestinal barrier structure, which is also a core issue restricting animal growth performance and nutrient utilization efficiency. This study found that after LPS modeling, the weight gain of mice was inhibited, the disease activity index (DAI) increased, the small intestine length shortened, and the intestinal villi and crypt structures were severely damaged (69); while CGA intervention could significantly improve these indicators, restore intestinal mucosal morphology, increase the ratio of villus height/duct depth (VH/CD), and effectively protect the integrity of the intestinal barrier structure and function (70).

This result provides direct experimental evidence for CGA’s role in improving livestock growth performance and maintaining intestinal health in animal nutrition regulation. LPS induces intestinal damage by inducing a large number of macrophage infiltration and exacerbating abnormal apoptosis of intestinal epithelial cells, manifested by significant increases in F4/80 and C-CAS3 expression. The experimental results show that CGA can significantly reduce the F4/80 positive area in small intestinal tissue, reduce inflammatory cell infiltration, and simultaneously down-regulate C-CAS3 levels to inhibit cell apoptosis, through anti-inflammatory and anti-apoptotic dual effects to alleviate LPS-induced intestinal damage. This optimization and repair effect on the intestinal microenvironment is of great practical significance for improving animal health levels and optimizing nutritional intervention plans.

Mechanism studies show that the PI3K inhibitor LY294002 can significantly reverse the protective effect of CGA, suggesting that the intestinal protective effect of CGA depends on the activation of the PI3K/Akt pathway. CGA can activate the PI3K/Akt-Nrf2/HO-1 signaling axis, enhance the body’s antioxidant and anti-inflammatory capabilities, thereby inhibiting inflammatory infiltration and cell apoptosis (71), and ultimately achieving the protective effect against LPS-induced intestinal damage in mice. The activation mechanism of this signaling axis provides key theoretical support for the application of CGA as a functional nutritional additive in animal nutrition regulation and functional feed development.

Despite the promising findings, several limitations remain. First, while functional activation of the Nrf2/HO-1 axis was confirmed via protein expression and pharmacological inhibition, direct detection of Nrf2 nuclear translocation was not conducted and will be addressed in future studies. Second, evaluating a single *in vivo* dose of CGA (100 mg/kg) limits our understanding of its dose–response efficacy. Third, the predicted interaction between CGA and Keap1 via molecular docking requires definitive experimental validation using techniques like surface plasmon resonance. Finally, although H₂O₂ (*in vitro*) and LPS (in vivo) represent distinct injury models, their shared convergence on ROS overproduction supports the broad applicability of CGA against oxidative stress, though specific upstream mechanistic nuances warrant further exploration.

## 5 Conclusion

In conclusion, CGA effectively alleviates oxidative stress and apoptosis in mouse small intestinal epithelial cells, and mitigates LPS-triggered intestinal damage in mice. Mechanistically, CGA competitively binds to Keap1 to activate the Nrf2/HO-1 pathway, and further modulates the PI3K/Akt signaling cascade. The PI3K/Akt-Nrf2/HO-1 signaling axis plays a pivotal role in mediating the intestinal protective function of CGA. Collectively, this evidence indicates that CGA can serve as a high-potential natural bioactive ingredient, and provides a theoretical basis for its practical application in animal intestinal health regulation and nutritional intervention.

## Data Availability

The original contributions presented in the study are included in the article/Supplementary material, further inquiries can be directed to the corresponding author/s.

## Footnotes

1
http://ctdbase.org/

2
https://www.genecards.org/

3
https://www.disgenet.org/

4
https://www.bioinformatics.com.cn
