# Supplementary material for: Protective effects of chlorogenic acid against LPS-induced intestinal oxidative injury in mice via activation of the PI3K/Akt-Nrf2/HO-1 signaling axis
Source: Front Vet Sci. 2026 Jul 10;13:1870702. doi: 10.3389/fvets.2026.1870702 (PMC13395676; doi:10.3389/fvets.2026.1870702)
Supplement: Supplementary file 1 [file Image_1.PDF]

吕梁学院动物实验伦理审查同意书
Affidavit of Approval of Animal Use Protocol

批准编号 Approval No.	LLXYLL20250401

本动物实验方案经过吕梁学院伦理委员会审核，符合动物保护、动物福利和伦理原则，符合国家实验动物福利伦理的相关规定。The animal use protocol listed below has been reviewed and approved by the Ethics Committee of Lyuliang University.

项目名称 Protocol Title	绿原酸对小鼠小肠损伤的保护作用及机制研究 Protective effect of chlorogenic acid on small intestinal injury in mice and its mechanism				
申请人 Applicant	贺莹 He Ying	职称/学位 Title/Degree	副教授/博士 Associate Professor/PhD	邮箱 Email	913267085@qq.com
负责人 Principle Investigator (PI)	贺莹 He Ying	职称/学位 Title/Degree	副教授/博士 Associate Professor/PhD	邮箱 Email	913267085@qq.com
院系(部门) Department	生物与食品工程系 Department of Biological and Food Engineering		申请日期 Application date		2025.04.10 April 10, 2025
动物种系 Species or Strains	小鼠 （C57BL/6） Mice （C57BL/6）		动物数量 Quantity		30 thirty
计划执行时间 Period of Protocol	2025.04.18-2025.09.18				
审查意见 Results of inspection	☑ 符合动物福利伦理要求，同意实验 **Agree**<br>☐ 调整方案后，可进行实验 **Agree after modification**				

吕梁学院伦理委员会

日期（Date）：2025.04.15

地址：山西省吕梁市离石区学院路1号 邮编：033001
Add.: Lyuliang University,
Xueyuan Road, Lishi District,
Lvliang, Shanxi Province,033001, P.R. China
