# Supplementary material for: Protective effects of chlorogenic acid against LPS-induced intestinal oxidative injury in mice via activation of the PI3K/Akt-Nrf2/HO-1 signaling axis
Source: Front Vet Sci. 2026 Jul 10;13:1870702. doi: 10.3389/fvets.2026.1870702 (PMC13395676; doi:10.3389/fvets.2026.1870702)
Supplement: Supplementary file 2 [file Image_2.PDF]

American Manuscript Editors

English Editing Certificate

This document certifies that the manuscript listed below was edited by the expert staff of American Manuscript Editors, all of whom are native English speakers. Moreover, the document was edited for proper English language, grammar, punctuation, and spelling.

Manuscript Title:

Protective Effects of Chlorogenic Acid Against LPS-Induced Intestinal Oxidative Injury in Mice via Activation of the PI3K/Akt-Nrf2/HO-1 Signaling Axis

Authors:

Ying He,Yuhan Wu,Yue Wei,Yuan Wang,Caiping Feng

Certificate Verification Key:

404-496-457-960-525

Project Number:

131921

This certificate may be verified by emailing info@americanmanuscripteditors.com. Documents receiving this certificate should be prepared for publication. However, please note that the author has the ability to accept or reject our suggestions for changes and can make changes after the editing process is complete, all of which can adversely affect the quality of the text after the editing process.

AMERICAN MANUSCRIPT EDITORS
