# Supplementary material for: Protective effects of chlorogenic acid against LPS-induced intestinal oxidative injury in mice via activation of the PI3K/Akt-Nrf2/HO-1 signaling axis
Source: Front Vet Sci. 2026 Jul 10;13:1870702. doi: 10.3389/fvets.2026.1870702 (PMC13395676; doi:10.3389/fvets.2026.1870702)
Supplement: Supplementary file 3 [file Table_1.docx]

Supplementary Material

Protective Effects of Chlorogenic Acid Against LPS-Induced Intestinal Oxidative Injury in Mice via Activation of the PI3K/Akt-Nrf2/HO-1 Signaling Axis

**Ying He^1*^, Yuhan Wu^1^, Yue Wei^1^, Yuan Wang^1^, Caiping Feng^1*^**

^1^Department of Biological and Food Engineering, Lyuliang University, Lishi, Shanxi, 033000, China

# Supplementary Data

# Supplementary Figures and Tables

## Supplementary Tables

**Table S1.**  Analysis of the topological properties of core targets

| **name** | **Betweenness Centrality** | **Closeness Centrality** | **Degree** | **Clustering Coefficient** | **Neighborhood Connectivity** | **Radiality** |
| --- | --- | --- | --- | --- | --- | --- |
| Hsp90aa1 | 0.17931033 | 0.49324324 | 20 | 0.27894737 | 10.2 | 0.85322896 |
| Kit | 0.10791024 | 0.48026316 | 18 | 0.2745098 | 9.44444444 | 0.84540117 |
| Fyn | 0.07426019 | 0.44512195 | 15 | 0.25714286 | 9.4 | 0.82191781 |
| Lck | 0.05218009 | 0.43975904 | 15 | 0.33333333 | 9.8 | 0.81800391 |
| Tlr4 | 0.13097154 | 0.47402597 | 15 | 0.2952381 | 9.93333333 | 0.84148728 |
| Maob | 0.13588701 | 0.42941176 | 13 | 0.08974359 | 4.92307692 | 0.81017613 |
| Dnmt1 | 0.06129021 | 0.41954023 | 13 | 0.32051282 | 9 | 0.80234834 |
| Braf | 0.07136763 | 0.45625 | 13 | 0.23076923 | 9.92307692 | 0.8297456 |
| Btk | 0.05874255 | 0.40782123 | 12 | 0.33333333 | 9.58333333 | 0.7925636 |
| Mcl1 | 0.04128335 | 0.41714286 | 11 | 0.29090909 | 10.36363636 | 0.80039139 |
| Tyms | 0.04391016 | 0.37055838 | 11 | 0.4 | 7.63636364 | 0.75733855 |
| Gart | 0.08672237 | 0.42196532 | 11 | 0.32727273 | 8 | 0.80430528 |
| Hdac1 | 0.06008384 | 0.41714286 | 11 | 0.45454545 | 9.18181818 | 0.80039139 |
| Ada | 0.07493768 | 0.38829787 | 10 | 0.37777778 | 7.4 | 0.77495108 |
| Dpp4 | 0.11684324 | 0.42941176 | 10 | 0.17777778 | 7.7 | 0.81017613 |
| Aurka | 0.02645099 | 0.41477273 | 10 | 0.44444444 | 11.1 | 0.79843444 |
| Dhfr | 0.02266552 | 0.39037433 | 9 | 0.52777778 | 9.11111111 | 0.77690802 |
| Cdk1 | 0.01242898 | 0.39673913 | 9 | 0.58333333 | 11.33333333 | 0.78277886 |
| Hdac6 | 0.00642985 | 0.38020833 | 9 | 0.63888889 | 9.66666667 | 0.76712329 |
| Prkcd | 0.00673645 | 0.38421053 | 8 | 0.39285714 | 10.875 | 0.77103718 |
| Mme | 0.03228677 | 0.3989071 | 8 | 0.32142857 | 8.375 | 0.78473581 |
| Ar | 0.00758362 | 0.40782123 | 8 | 0.67857143 | 12.875 | 0.7925636 |
| Ahcy | 0.00599112 | 0.34761905 | 7 | 0.66666667 | 9 | 0.73189824 |
| Adora2a | 0.05644722 | 0.38624339 | 7 | 0.33333333 | 7 | 0.77299413 |
| Cd22 | 0.00496238 | 0.37244898 | 7 | 0.57142857 | 11.28571429 | 0.7592955 |
| Casp1 | 0.0183675 | 0.39459459 | 7 | 0.33333333 | 10 | 0.78082192 |
| Prkca | 0.02276215 | 0.40331492 | 7 | 0.14285714 | 9.28571429 | 0.78864971 |
| Sell | 0.00466748 | 0.36138614 | 7 | 0.57142857 | 11 | 0.74755382 |
| Htr2c | 0.03103845 | 0.36868687 | 7 | 0.19047619 | 6.14285714 | 0.7553816 |
| Flt1 | 0.02659942 | 0.44242424 | 7 | 0.57142857 | 14.57142857 | 0.81996086 |
| Ptpn1 | 0.01459752 | 0.37435897 | 6 | 0.2 | 8.5 | 0.76125245 |
| Bace1 | 0.02561108 | 0.41011236 | 6 | 0.2 | 9.33333333 | 0.79452055 |
| Pik3cg | 0.01900856 | 0.38624339 | 6 | 0.4 | 11.33333333 | 0.77299413 |
| Prkcq | 0.00227951 | 0.36868687 | 6 | 0.6 | 11.66666667 | 0.7553816 |
| Csnk2a1 | 0.0107437 | 0.37823834 | 6 | 0.6 | 10.83333333 | 0.76516634 |
| Impdh2 | 0.00204753 | 0.33181818 | 5 | 1 | 9.6 | 0.71232877 |
| Cda | 0.00251721 | 0.34433962 | 5 | 0.7 | 10.8 | 0.72798434 |
| Adora2b | 0.00826249 | 0.30672269 | 5 | 0.5 | 5.4 | 0.67710372 |
| Ehmt2 | 9.3455E-4 | 0.33181818 | 5 | 0.7 | 8.4 | 0.71232877 |
| Csnk2a2 | 0.05403349 | 0.36318408 | 5 | 0.6 | 9.6 | 0.74951076 |

**Table S2. KEGG pathway enrichment analysis of core targets**

| Pathway ID | Description | P value | Gene_names |
| --- | --- | --- | --- |
| mmu04726 | Serotonergic synapse | 5.19E-05 | HTR7, MAOB, ALOX5, HTR2C, BRAF, PRKCA, PTGS1, SLC6A4 |
| mmu04270 | Vascular smooth muscle contraction | 9.46E-05 | EDNRA, PRKCH, ADORA2A, ADORA2B, PRKCD, BRAF, PRKCQ, PRKCA |
| mmu04020 | Calcium signaling pathway | 5.69E-04 | CHRM2, EDNRA, FLT1, HTR7, ADORA2A, ADORA2B, P2RX1, HTR2C, PRKCA |
| mmu04064 | NF-kappa B signaling pathway | 0.001101835 | CSNK2A1, LCK, CSNK2A2, BTK, PRKCQ, TLR4 |
| mmu01523 | Antifolate resistance | 0.001278306 | DHFR, FPGS, TYMS, GART |
| mmu04151 | PI3K-Akt signaling pathway | 0.00134452 | CHRM2, HSP90AA1, FLT1, INSR, KIT, PRKCA, JAK3, TLR4, PIK3CG, MCL1 |
| mmu05031 | Amphetamine addiction | 0.001727955 | GRIA1, MAOB, HDAC1, PRKCA, SLC6A3 |
| mmu05340 | Primary immunodeficiency | 0.0024061 | LCK, BTK, JAK3, ADA |
| mmu05235 | PD-L1 expression and PD-1 checkpoint pathway in cancer | 0.00419084 | CSNK2A1, LCK, CSNK2A2, PRKCQ, TLR4 |
| mmu05034 | Alcoholism | 0.004220791 | MAOB, ADORA2A, ADORA2B, HDAC1, BRAF, HDAC6, SLC6A3 |
| mmu04520 | Adherens junction | 0.004910694 | PTPN1, CSNK2A1, INSR, CSNK2A2, FYN |
| mmu04015 | Rap1 signaling pathway | 0.005199289 | FLT1, ADORA2A, ADORA2B, INSR, KIT, BRAF, PRKCA |
| mmu04080 | Neuroactive ligand-receptor interaction | 0.008514979 | CHRM2, GRIA1, EDNRA, HTR7, ADORA2A, ADORA2B, P2RX1, ADORA1, HTR2C |
| mmu00670 | One carbon pool by folate | 0.0087087 | DHFR, TYMS, GART |
| mmu04923 | Regulation of lipolysis in adipocytes | 0.00881741 | INSR, ADORA1, MGLL, PTGS1 |
| mmu01100 | Metabolic pathways | 0.013046273 | CDA, DNMT1, AHCY, MAOB, EHMT2, GAA, KMO, TYMS, TYR, PIK3CG, PTGS1, DHFR, DAO, ALOX5, IMPDH2, ANPEP, FPGS, LTA4H, GART, MGLL, ADA |
| mmu04664 | Fc epsilon RI signaling pathway | 0.013147457 | ALOX5, BTK, FYN, PRKCA |
| mmu04611 | Platelet activation | 0.014175101 | P2RX1, BTK, FYN, PIK3CG, PTGS1 |
| mmu04750 | Inflammatory mediator regulation of TRP channels | 0.01494922 | PRKCH, PRKCD, HTR2C, PRKCQ, PRKCA |
| mmu05200 | Pathways in cancer | 0.019340179 | AR, EDNRA, HSP90AA1, HDAC1, KIT, ABL1, HES1, BRAF, PRKCA, JAK3 |
| mmu04613 | Neutrophil extracellular trap formation | 0.019556573 | SELP, HDAC1, CASP1, PRKCA, TLR4, HDAC6 |
| mmu01232 | Nucleotide metabolism | 0.024896111 | CDA, IMPDH2, TYMS, ADA |
| mmu04072 | Phospholipase D signaling pathway | 0.02525654 | INSR, KIT, FYN, PRKCA, PIK3CG |
| mmu04024 | cAMP signaling pathway | 0.025956181 | CHRM2, GRIA1, EDNRA, ADORA2A, ADORA1, BRAF |
| mmu04014 | Ras signaling pathway | 0.031057301 | FLT1, HTR7, INSR, KIT, ABL1, PRKCA |
| mmu04914 | Progesterone-mediated oocyte maturation | 0.031461466 | HSP90AA1, CDK1, BRAF, AURKA |
| mmu04640 | Hematopoietic cell lineage | 0.033231519 | MME, ANPEP, KIT, CD22 |
| mmu04659 | Th17 cell differentiation | 0.043874318 | HSP90AA1, LCK, PRKCQ, JAK3 |
| mmu04931 | Insulin resistance | 0.049210807 | PTPN1, INSR, PRKCD, PRKCQ |

### Table S3. ****Effects of different concentrations of H₂O₂ on the viability of MODE-K cells****

| **H₂O₂ concentration (μmol/L)** | **Viable Cell Concentration (×10⁴ cells/mL)** | **Relative cell viability (%)** |
| --- | --- | --- |
| **0 (Control)** | 9.93 ± 0.37 | 100.0 ± 3.72 |
| **50** | **10.85 ± 0.35** | 109.3 ± 3.48 |
| **100** | **6.58 ± 0.26** | 66.3 ± 2.63 |
| **150** | **4.66 ± 0.27** | **46.91 ± 2.73** |
| **200** | **3.64 ± 0.34** | 36.68 ± 3.41 |
| **400** | **1.52 ± 0.03** | 15.3 ± 0.30 |

### Table S4. ****Protective effect of CGA on H₂O₂ -induced MODE-K cell damage****

| **Experimental Groups** | **Viable Cell Concentration (×10⁴ cells/mL)** | **Relative cell viability (%)** |
| --- | --- | --- |
| **Control** | **9.80 ± 0.23** | 100.00 ± 2.31 |
| **Model** | **4.19 ± 0.25** | 42.84 ± 2.67 |
| **CGA (12.5 μmol/L)** | **4.13 ± 0.05** | 42.12 ± 0.53 |
| **CGA (25 μmol/L)** | **5.78 ± 0.67** | 58.97 ± 6.82 |
| **CGA (50 μmol/L)** | **8.14 ± 0.19** | 83.05 ± 1.94 |
| **CGA (100 μmol/L)** | **9.60 ± 0.33** | 97.98 ± 3.4 |
| **CGA (200 μmol/L)** | **9.49 ± 0.45** | 96.82 ± 4.6 |
